# Constructing Novel 2D Composite Nanomaterials by Coupling Graphene or Silicene with TM_3_N_2_ MXene (TM = Nb, Ta, Mo, and W) to Achieve Highly Efficient HER Catalysts

**DOI:** 10.3390/molecules30112401

**Published:** 2025-05-30

**Authors:** Xiuyi Zhang, Guangtao Yu, Wei Zhang, E Yang, Wei Chen

**Affiliations:** 1 Engineering Research Center of Industrial Biocatalysis, Fujian Provincial Key Laboratory of Advanced Materials Oriented Chemical Engineering, Fujian-Taiwan Science and Technology Cooperation Base of Biomedical Materials and Tissue Engineering, College of Chemistry and Materials Science, Fujian Normal University, Fuzhou 350007, China; sheazhangstart@163.com (X.Z.); yangeli66@fjnu.edu.cn (E.Y.); 2 Academy of Carbon Neutrality of Fujian Normal University, Fuzhou 350007, China; 3 Fujian Provincial Key Laboratory of Quantum Manipulation and New Energy Materials, College of Physics and Energy, Fujian Normal University, Fuzhou 350117, China; 4 Fujian Provincial Key Laboratory of Theoretical and Computational Chemistry, Xiamen University, Xiamen 361005, China

**Keywords:** TM_3_N_2_ MXenes, graphene/silicene, electrocatalysts, hydrogen evolution reaction (HER), DFT calculations

## Abstract

MXenes have emerged as promising candidates for energy storage and catalyst design. Through detailed density functional theory (DFT) calculations, we designed a series of new 2D composite MXene-based nanomaterials by covering excellent TM_3_N_2_ MXenes (TM = Nb, Ta, Mo, and W) with graphene or buckled silicene. Our findings demonstrate that this coating can lead to high catalytic activity for hydrogen evolution reactions (HER) in these composite MXene-based systems, with silicene exhibiting superior performance compared to graphene. The relevant carbon and silicon atoms in the coated materials serve as active sites for HER due to complex electron transfer processes. Additionally, doping N or P atoms into graphene/silicene, which have similar atomic radii, but larger electronegativity than C/Si atoms, can further enhance the HER activity of adjacent carbon or silicon atoms, thus endowing the composite systems with higher HER catalytic performance. Coupled with their high stability and metallic conductivity, all these composite systems show great potential as electrocatalysts for HER. These remarkable findings offer new strategies and valuable insights for designing non-precious and highly efficient MXene-based HER electrocatalysts.

## 1. Introduction

The emissions of greenhouse gases from fossil fuels have intensified the phenomenon of global warming, raising substantial concerns regarding the future state of our environment [1,2,3]. Transitioning from finite fossil fuel resources to renewable green energy sources is imperative for ensuring the sustainability of our future [4]. Hydrogen, recognized as a clean and renewable energy carrier, has garnered significant attention owing to its high energy density and absence of pollution [5,6,7]. Among the various methodologies for hydrogen production, electrochemical water splitting emerges as one of the most promising approaches and has been extensively researched [8]. However, as one of the half reactions in water electrolysis, the hydrogen evolution reaction (HER, 2H^+^ + 2e^−^ = H_2_) is a slow kinetic process, so it is necessary to use effective HER catalysts to minimize the overpotential [9,10]. Platinum-based materials are widely recognized as the most effective HER electrocatalysts [10]; however, their high cost and limited availability restrict their widespread adoption. To overcome this barrier, researchers are dedicating substantial efforts to finding alternative materials that can offer high efficiency in HER catalysis.

Presently, research into HER catalysts derived from two-dimensional (2D) materials is garnering significant attention [11,12,13,14,15,16,17,18,19,20,21,22]. This primarily arises from the inherent advantages of 2D nanomaterials, which provide a larger specific surface area and a greater number of accessible active sites [23,24], thereby facilitating the interaction between reactants and active sites. Theoretical and experimental investigations have demonstrated that various types of 2D materials can exhibit good HER activity, such as transition metal compounds [25,26,27,28], layered double hydroxides (LDHs) [29,30], metal–organic frameworks (MOFs) [31,32], and graphene-based materials [33,34].

Among others, MXenes, a significant class of 2D materials, have emerged as promising candidates for HER electrocatalysis [35]. They belong to the family of transition metal carbides, nitrides, and carbonitrides, with the general chemical formula M_n+1_X_n_, where M represents transition metal elements, X is carbon or nitrogen, and n is 1, 2, or 3. MXenes can exhibit a range of unique advantages [36,37,38], including high electrical conductivity, tunable surface chemistry, excellent gravimetric capacitance, favorable hydrophilicity, and adjustable mechanical properties. These attributes make MXenes highly versatile for various applications, particularly in electrocatalysis. Carbon-containing MXenes (C-MXenes) have been widely investigated for HER electrocatalysis. For instance, the Mo_2_CT_x_ MXene has demonstrated commendable HER catalytic performance in both experimental and theoretical studies [39]. Additionally, Cr_2_CT_x_, synthesized using LiF and H_2_SO_4_ as etchants, exhibits both efficient HER activity coupled with high stability [40]. Moreover, Li et al. developed F-terminated Ti_2_CT_x_ nanosheets, which feature low initiation overpotentials and a high density of active sites for HER [41].

In contrast, research on N-containing MXenes for HER catalysis remains relatively scarce. However, transition metal nitrides (TMNs) can be regarded as ideal electrocatalysts due to their excellent thermochemical stability, electrical conductivity, and catalytic efficiency [42]. The bonding with N atoms can regulate the *d*-band structure of the host metal, thereby narrowing the *d*-band width. This adjustment can make their electronic structures resemble that of noble metal Pt [43], endowing potential HER catalytic performance. Furthermore, the concept of the superatom—a stable assembly of atoms that mimics the chemical behavior of an elemental atom—provides a promising avenue for designing non-precious atomic clusters to model noble elements. For instance, the diatomic molecular ions NbN^−^/ZrO^−^ and WC^−^ can exhibit superatomic electronic structures similar to those of the noble metal ions Pd^−^ and Pt^−^, respectively [44,45]. Additionally, the triatomic anion HNbN^−^ shares a similar electronic structure with the Pt atom, enabling it to exhibit comparable reactivities in activating C–H bonds in methane and ethane [46]. Therefore, utilizing the superatom concept may facilitate the design of non-precious metal HER catalysts with high activity comparable to that of the relevant noble metals.

Inspired by the aforementioned considerations, our focus was on a series of 2D N-containing TM_3_N_2_ (TM = Nb, Ta, Mo, and W) MXenes selected from the Computational 2D Materials Database (C2DB) [47]. The objective of this work was to design composite systems that exhibit high catalytic performance for HER based on these four intriguing TM_3_N_2_ MXenes, in which the unique diatomic combination of NbN and WC, as well as their equivalent electronic analogues TaN, MoC, WSi, and MoSi, will be included. Specifically, we initially designed a series of new TM_3_N_2_/G composite structures by coupling graphene with the four TM_3_N_2_ MXenes, considering the remarkable chemical stability and superior electrical conductivity of graphene [48,49]. It was highly anticipated that coating graphene onto these TM_3_N_2_ MXenes could lead to high HER catalytic activity. Additionally, we propose another new strategy through covering wrinkled silicene to achieve high HER catalytic performance in TM_3_N_2_/Si composite systems. Silicene, regarded as an analogue of graphene, features relatively weaker π bonds between adjacent Si atoms compared to the robust π bonds in graphene. This characteristic could facilitate the interaction of silicene with TM_3_N_2_ MXenes through *sp*^3^ hybridization, thereby promoting HER catalytic activity on the silicene surface within the composite system. Furthermore, we incorporated N/P atoms, which have atomic radii similar to those of C/Si atoms, but exhibit greater electronegativity, into graphene and silicene of the composite systems. The introduction of N and P atoms is expected to induce a complex electron transfer process within the composite structures, effectively modulating the electron density on the surface of graphene/silicene, thus achieving higher HER activity. Overall, this work provides some new and effective strategies for designing highly efficient HER electrocatalysts based on the excellent MXene materials.

## 2. Results and Discussion

### 2.1. Structures, Stability, and Electronic Properties of 2D TM_3_N_2_ (TM = Nb, Ta, Mo, and W) MXene Systems

**Figure 1:**
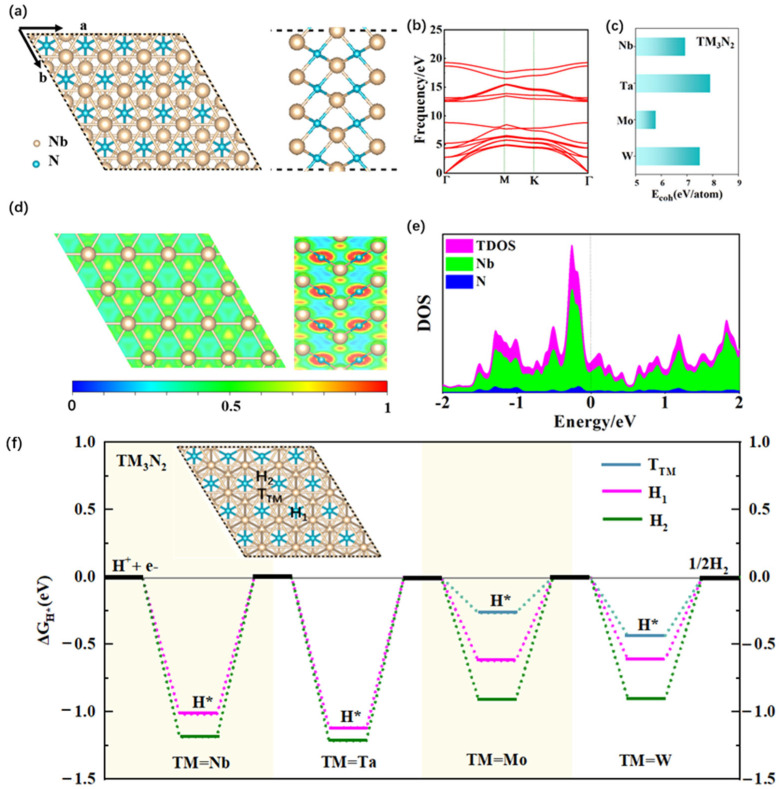
(**a**) Top and side views of the optimized structure for the Nb_3_N_2_ monolayer consisting of five atomic layers arranged in a stacking mode of TM–N–TM–N–TM (from left to right). (**b**) The corresponding phonon spectra. (**c**) The cohesion energies of TM_3_N_2_ (TM = Nb, Ta, Mo, and W) monolayers. (**d**) ELF maps, where the red regions with an ELF value around 1 indicate areas of high electron density; the green areas with an ELF value around 0.5 represent a homogeneous electron gas; and the blue regions with an ELF value near 0 mean areas of low electron density. (**e**) DOS of the Nb_3_N_2_ monolayer. (**f**) The ΔG_H*_ values of different adsorption sites on the TM_3_N_2_ surface. The data correspond to conditions of 1 bar of H_2_ at 298 K.

Initially, we conducted structural optimization for four 2D TM_3_N_2_ (TM = Nb, Ta, Mo, and W) MXene systems. The optimized geometrical structures are depicted in Figure 1a and Figure S1, all of which belong to the P-6m^2^ space group. It can be observed that these TM_3_N_2_ monolayers can consist uniformly of five atomic layers arranged in a TM–N–TM–N–TM stacking mode. Subsequently, phonon dispersion calculations for the four monolayers are performed within the first Brillouin zone along the high-symmetry K-point direction(Γ-M-K-Γ), as shown in Figure 1b and Figure S1. The unit cell of each TM_3_N_2_ monolayer can include five atoms. Thus, a total of fifteen phonon modes can be observed in their phonon spectra, including three acoustic branches and twelve optical branches. As illustrated in Figure 1b and Figure S1, there are no imaginary phonon modes within the first Brillouin zone for these TM_3_N_2_ monolayers, and all the phonon modes are positive, confirming their dynamic stability. The phonon frequency range for these four TM_3_N_2_ monolayers is approximately 0−20 eV for TM = Nb and Ta, and 0–17 eV for TM = Mo and W, respectively. The calculated lattice constants for Nb_3_N_2_, Ta_3_N_2_, Mo_3_N_2_, and W_3_N_2_ are a = b = 2.945 Å, 2.882 Å, 2.830 Å and 2.738 Å, respectively (Table S1). The calculated bond lengths of Nb–Nb, Ta–Ta, Mo–Mo, and W–W can be 2.945, 2.882, 2.830 and 2.738 Å, respectively, while. the TM–N bond lengths can fall within the range of 2.205–2.209 Å, 2.189–2.208 Å, 2.147–2.172 Å, and 2.130–2.264 Å for TM = Nb, Ta, Mo, and W, respectively. Our computed lattice constants and bond lengths for these four TM_3_N_2_ monolayers are well consistent with the corresponding results from the Computational 2D Materials Database (C2DB) [47]. It is worth mentioning that all these TM-TM and TM-N bond lengths are comparable to those in the experimentally synthesized systems (Table S2), indicating that these four TM_3_N_2_ monolayers could exhibit high structural stability.

The high stability of these monolayers can be further evidenced by their large cohesion energies (E_coh_), calculated using Formula (1). The E_coh_ values for the Nb_3_N_2_, Ta_3_N_2_, Mo_3_N_2_, and W_3_N_2_ monolayers can be as large as 6.93, 7.91, 5.78, and 7.50 eV/atom, respectively, indicating their high thermodynamic stability (Figure 1c and Table S1). These values can be comparable to or even larger than those of many typical 2D structures that have been successfully synthesized in the experiments, such as silicene (3.98) [50], germanene (3.26) [50], Cu_2_Si (3.46) [50], phosphorene (3.30) [51], MoS_2_ (3.59) [52], MoTe_2_ (2.05) [52], Ti_2_C MXenes (6.12) [53], β-Te (2.59) [54], and Mo_2_C (7.40 eV/atom) [55]. It is highly anticipated that these four 2D TM_3_N_2_ monolayers can be realized experimentally in the near future.

Furthermore, electron location function (ELF) calculations were conducted to analyze the nature of the related bonds in the TM_3_N_2_ monolayers (TM = Nb, Ta, Mo, and W) by slicing the corresponding planes containing the TM-TM and TM-N bonds, as shown in Figure 1d and Figure S1. A calculated ELF value of approximately 0.5 can be observed between two adjacent TM atoms, implying the presence of a metallic bond between them. A noticeable difference in ELF is observed around the TM and N atoms, indicating that the TM-N bond exhibits ionic character. This can be further supported by the Bader charge population analysis, which reveals a transfer of approximately 1.25–1.46 |e| electrons from TM to N atoms in the TM_3_N_2_ systems (Table S3). These strong chemical bonds can be crucial contributors to their high stability.

In addition, we evaluated the mechanical stability for the four TM_3_N_2_ monolayers by calculating their elastic constants using the finite difference method [56]. As shown in Table S4, all the calculated elastic constants can meet the Born criteria for 2D hexagonal material (i.e., C_11_C_22_ > C_12_^2^ and C_11_, C_22_, C_66_ > 0), indicating that these four TM_3_N_2_ monolayers can uniformly possess high mechanical stability. We also investigate the electronic property of the four TM_3_N_2_ monolayers by calculating their density of states (DOSs), as shown in Figure 1e and Figure S1. The calculated DOS results reveal that the correlated states, mainly originating from TM atoms, can pass through the Fermi level, indicating metallic conductivity in these four monolayers. It is well known that metallic conductivity facilitates rapid electron transfer, thereby enhancing the activity and efficiency of catalysts.

Clearly, all four MXene monolayers of TM_3_N_2_ (TM = Nb, Ta, Mo, and W) can uniformly exhibit high stability and excellent conductivity. This can provide a crucial theoretical foundation for designing new non-precious and highly efficient HER electrocatalysts based on these MXene systems.

Subsequently, we investigated the HER catalytic activity of these four 2D MXene systems by calculating the free energy (ΔG_H*_) of individual hydrogen atom adsorption on their surfaces. It is well known that the ΔG_H*_ value at a site can serve as an effective descriptor for qualitatively evaluating its HER catalytic activity [10], as HER activity is closely correlated with the adsorption energy of a single H atom. Usually, a smaller absolute value of ΔG_H*_ signifies better HER catalytic activity. It is worth mentioning that the descriptor ΔG_H*_ has been widely employed to predict the HER catalytic performance of various material systems [57,58,59,60,61,62,63,64].

Specifically, we calculated the ΔG_H*_ values to evaluate the HER catalytic activity of the four 2D TM_3_N_2_ systems (TM = Nb, Ta, Mo, and W), where all four possible adsorption sites of H* were considered, including one top site over the TM atom (T_TM_), one bridge site over the TM-TM bond (B_TM-TM_), and two hollow sites over the three-membered rings composed of TM atoms with or without the corresponding N atom positioned beneath the rings’ center (denoted H_1_ and H_2_, respectively). Our computed results reveal that the ΔG_H*_ value at the T_Mo_ site on Mo_3_N_2_ is relatively small at −0.266 eV (Figure 1f and Table S5), indicating good HER catalytic activity at this site. In contrast, the remaining adsorption sites H_1_ (−0.622) and H_2_ (−0.903 eV) on Mo_3_N_2_ exhibit large absolute values of ΔG_H*_, suggesting poor HER activity. Unlike 2D Mo_3_N_2_, the other three systems including W_3_N_2_, Nb_3_N_2_ and Ta_3_N_2_ can present relatively weak or inert catalytic activity for HER, due to their relatively large negative ΔG_H*_ values ranging from −0.435 to −1.212 eV at the relevant adsorption sites (T_TM_, H_1_ or H_2_), as shown in Figure 1f and Table S5.

Obviously, among these four 2D MXene systems, only the Mo_3_N_2_ monolayer can exhibit relatively high HER catalytic performance. Considering the largely negative ΔG_H*_ values of H* on nearly all adsorption sites of these four TM_3_N_2_ monolayers, we propose new strategies through covering 2D graphene or silicene to design the highly efficient HER electrocatalysts based on these excellent MXene systems in the subsequent sections.

### 2.2. Structures and HER Catalytic Performance of New 2D Composite MXenes/G or MXenes/NG Systems by Coupling the TM_3_N_2_ (TM = Nb, Ta, Mo or W) Monolayers with Pure Graphene or N-Doped Graphene

#### 2.2.1. Structures and HER Catalytic Activity of 2D Composite TM_3_N_2_/G Systems

In order to improve the HER catalytic activity of the four 2D MXene systems, we designed novel 2D composite structures by coupling the TM_3_N_2_ (TM = Nb, Ta, Mo or W) monolayer with graphene. Graphene, an important member of the 2D carbon-based material family, features a large area of delocalized π-conjugated skeleton composed of six-membered carbon rings, leading to outstanding thermal, mechanical and electrical properties [48,65]. It is highly anticipated that these new 2D composite nanostructures, created by combining MXenes with graphene, can exhibit high stability and excellent conductivity. Additionally, effective charge transfer may occur between the TM-metal layer and graphene in the composite systems, potentially leading to the formation of highly active HER catalytic sites. Particularly, it is worth mentioning the atomic combination of WC can be regarded as the superatom corresponding to the noble metal Pt [45]. Therefore, the interaction between the W and C atoms produced by covering the W_3_N_2_ monolayer with graphene could induce considerably high or even Pt-like HER catalytic activity in the composite system. For convenience, these 2D composite systems are labeled TM_3_N_2_/G.

**Figure 2:**
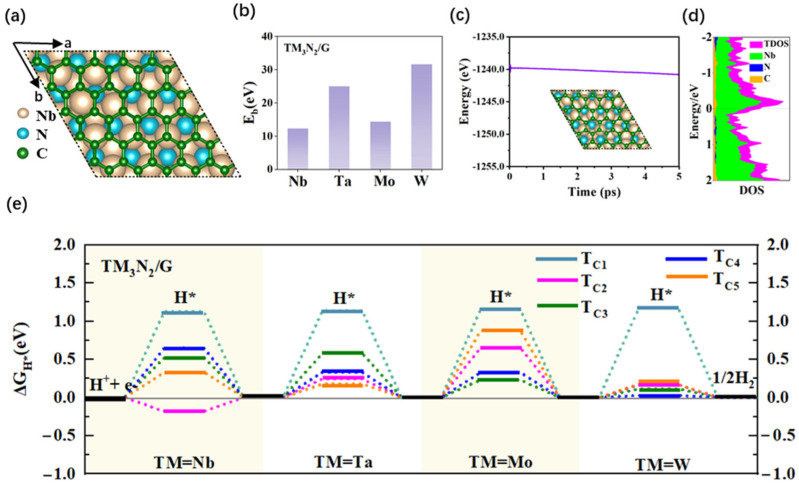
(**a**) Top views of the optimized structure for the Nb_3_N_2_/G nanostructure. (**b**) The binding energies of TM_3_N_2_/G (TM = Nb, Ta, Mo, and W) systems. (**c**) The AIMD simulation for Nb_3_N_2_/G nanostructure at 500 K. Insets: snapshot of the structure after 5 *ps*. (**d**) The DOS of the Nb_3_N_2_/G nanostructure, where the Fermi level is set to zero. (**e**) The ΔG_H*_ values of different adsorption sites (refer to Figure S2) on the surface of TM_3_N_2_/G systems. The data correspond to conditions of 1 bar of H_2_ at 298 K.

As shown in Figure 2a and Figure S2, four composite TM_3_N_2_/G (TM = Nb, Ta, Mo, and W) nanostructures can be obtained by covering the corresponding MXene monolayers with graphene. The calculated lattice parameters a/b for these TM_3_N_2_/G systems are in the range of 12.139–16.783 Å (Table S6). Furthermore, we calculated the binding energy (E_b_) of TM_3_N_2_/G using Formula (2) to evaluate their structural stability (Figure 2b and Table S6). The calculated results show that all of the composite systems can exhibit large E_b_ values in the range of 12.344–31.689 eV per unit cell (Table S6). This suggests that depositing graphene on these four MXene monolayers can be a favorable energy process, and the resulting composite nanostructures can possess high structural stability.

Subsequently, we investigated the HER catalytic activity of the four 2D composite TM_3_N_2_/G (TM = Nb, Ta, Mo, and W) systems by calculating the ΔG_H*_ values. All potential adsorption sites on the graphene surface within the composite systems were considered, and a total of five representative top sites over the C atoms was eventually obtained for each TM_3_N_2_/G nanostructure (Figure S2), including the carbon atoms over the TM atom, a three-membered ring composed of TM atoms with the corresponding N atom positioned beneath the ring’s center, a three-membered ring composed of TM atoms without the N atom beneath the ring’s center, the TM-TM bond, and the TM-N bond. For convenience, these five sites are denoted T_C1_, T_C2_, T_C3_, T_C4_ and T_C5_, respectively.

Initially, we explored the HER catalytic activity of W_3_N_2_/G by calculating ΔG_H*_ values. Our calculated results revealed that the ΔG_H*_ values for all the four adsorption sites T_C2_–T_C5_ can be as small as 0.168, 0.112, 0.028 and 0.170 eV, respectively (Figure 2e and Table S8), indicating considerably high HER catalytic activity at these sites. Among them, the T_C4_ site (0.028 eV) was identified as the most active site, given its near-zero ΔG_H*_ values. In contrast, the remaining T_C1_ site (1.176 eV) can have large ΔG_H*_ value, suggesting poor HER catalytic activity at this site. Clearly, compared with pure 2D MXene W_3_N_2_, the composite W_3_N_2_/G system formed by coupling with graphene demonstrates high HER catalytic activity, where most carbon atoms can serve as active sites for HER.

As for the analogous Mo_3_N_2_/G system, which contains Mo metal atom in the same main group as the W atom, the calculated ΔG_H*_ value for the T_C3_ site can be as small as 0.231 eV (Figure 2e and Table S8), suggesting high HER catalytic activity. Additionally, the T_C4_ site also exhibits good HER activity, due to a relatively small ΔG_H*_ value of 0.330 eV. The remaining three sites, including T_C1_ (1.155), T_C2_ (0.653) and T_C5_ (0.876 eV), have large ΔG_H*_ values, indicating poor or relatively weak catalytic activity for HER. Obviously, in contrast to pure Mo_3_N_2_ monolayer, the 2D composite Mo_3_N_2_/G system can exhibit higher HER catalytic activity, where the carbon atoms at the T_C3_ site can serve as the most active sites.

A similar improvement in HER performance can also be observed in the 2D composite Nb_3_N_2_/G system. Specifically, the T_C2_ site (−0.182 eV) can be employed as the most active site, and the T_C5_ site (0.332 eV) also displays good HER activity, as presented in Figure 2e and Table S8. The remaining three sites, T_C1_ (1.128), T_C3_ (0.523) and T_C4_ (0.649 eV), exhibit relatively weak or inert HER activity, due to their relatively large ΔG_H*_ values. Comparatively, the analogous Ta_3_N_2_/G system, containing Ta metal atom in the same main group as Nb, can demonstrate even higher HER catalytic activity. Specifically, two sites on Ta_3_N_2_/G display very small ΔG_H*_ values (Figure 2e and Table S8), namely the T_C2_ (0.243 eV) and T_C5_ (0.179 eV) sites, indicating considerably high HER catalytic activity. In addition, the T_C4_ site can also exhibit good HER activity, due to a relatively small ΔG_H*_ value of 0.330 eV. The remaining T_C1_ (1.138) and T_C3_ (0.581) sites exhibit poor or relatively weak catalytic activity for HER.

Furthermore, we also conducted AIMD simulations to evaluate the thermal stability of 2D TM_3_N_2_/G (TM = Nb, Ta, Mo, and W) systems, which lasted for 5 *ps*, with a time step of 1 *fs*, at a constant temperature of 500 K. As shown in Figure 2c and Figure S3, their total free energies only exhibit slight fluctuations around a constant value throughout the simulation process, and the monolayer structures are well maintained with almost no structural deformation, indicating high thermal stability. In addition, metallic behavior can also be observed for all four TM_3_N_2_/G (TM = Nb, Ta, Mo, and W) systems, where the relevant states crossing the Fermi level mainly originate from TM atoms (Figure 2d and Figure S4).

Obviously, all these new 2D TM_3_N_2_/G (TM = Nb, Ta, Mo, and W) systems, especially W_3_N_2_/G, can uniformly exhibit high HER catalytic activity, with the relevant C atoms on the subunit graphene serving as the active sites. Covering graphene onto 2D MXene monolayers containing TM/N components can be regarded as an effective strategy for achieving non-precious and highly efficient HER electrocatalysts.

#### 2.2.2. Further Enhancing the Catalytic Activities for HER on 2D Composite TM_3_N_2_/G Structures by Doping N Atoms into Graphene

Based on the findings above, we can understand that compared to the corresponding TM_3_N_2_ monolayers, the 2D composite TM_3_N_2_/G (TM = Nb, Ta, Mo, and W) systems exhibit high HER catalytic activity. However, it is anticipated that the HER activity of these composite systems can be further improved by reducing positive ΔG_H*_ values of the relevant sites. In this study, we propose an effective strategy to further optimize the adsorption state of H* on TM_3_N_2_/G by doping N atoms into the graphene subunit, considering that N atoms have an atomic radius similar to that of C atoms, but possess greater electronegativity.

Initially, we constructed 2D composite TM_3_N_2_/NG (TM = Nb, Ta, Mo, and W) nanostructures by covering N-doped graphene onto the TM_3_N_2_ monolayer (Figure S6), which can be regarded as the doped MXenes/G system with N at the C site on the graphene. Our computed results show the lattice parameters a/b of these doped TM_3_N_2_/NG systems fall within the range of 12.129–16.777 Å (Table S6), which are comparable to the corresponding pristine TM_3_N_2_/G systems (12.139–16.783 Å), indicating the negligible effect of N-doping on the overall structures. Furthermore, the calculated binding energy E_b_ values for these doped TM_3_N_2_/NG systems can be as large as 12.808–31.653 eV per unit cell (Figure S5 and Table S6), all of which are comparable to those of the corresponding pristine TM_3_N_2_/G systems (12.344–31.689 eV), confirming their high structural stability as well.

**Figure 3:**
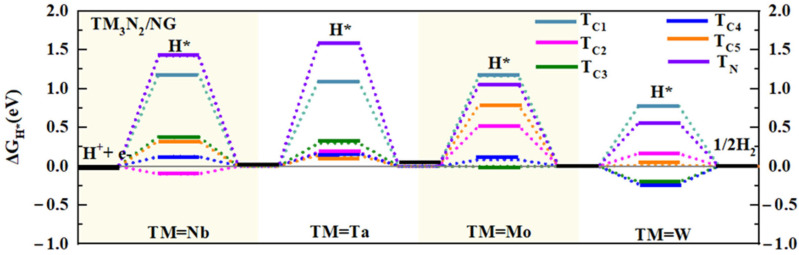
ΔG_H*_ values of different adsorption sites (refer to Figure S6) on the surface of TM_3_N_2_/NG (TM = Nb, Ta, Mo, and W) systems. The data correspond to conditions of 1 bar of H_2_ at 298 K.

Subsequently, we explored the impact of doping N atoms on the HER catalytic activity of the 2D composite TM_3_N_2_/G (TM = Nb, Ta, Mo, and W) systems by calculating the ΔG_H*_ values, where all five representative carbon sites (T_C1_–T_C5_) and the doped N site (T_N_) are considered, as illustrated in Figure S6. Specifically, when doping N atoms into the subunit graphene in Nb_3_N_2_/G, the calculated ΔG_H*_ value for the T_C2_ site can be changed from −0.182 to −0.101 eV (Figure 3 and Table S8), indicating an enhancement in HER catalytic activity. Consequently, the T_C2_ site remains the most active site. The HER catalytic activity of the T_C4_ site is significantly improved, as evidenced by the reduction in its ΔG_H*_ value from 0.649 to 0.124 eV. Additionally, the ΔG_H*_ value of the T_C3_ site can also decrease from 0.523 to 0.381 eV, endowing the T_C3_ site with certain HER activity. Moreover, the remaining T_C5_ (0.325 eV) site can also exhibit certain HER activity, while the T_C1_ (1.182) and T_N_ (1.423 eV) sites continue to show poor HER performance due to its large ΔG_H*_ value. Clearly, doping N atoms can effectively enhance the HER catalytic activity of Nb_3_N_2_/G, endowing the composite Nb_3_N_2_/NG system with higher HER performance.

A similar situation can be observed in the analogous N-doped Ta_3_N_2_/NG system (Figure 3 and Table S8). It was found that the doping of N atoms can effectively decrease the corresponding ΔG_H*_ values for the T_C2_, T_C4_ and T_C5_ sites from their original values of 0.243, 0.330 and 0.179 eV to 0.176, 0.169 and 0.141 eV, respectively, indicating an enhancement in their HER catalytic activities. It is evident that all three sites can exhibit considerably high HER activity due to the very small ΔG_H*_ values. Additionally, the introduction of N atoms also enhances the HER activity of the T_C3_ site by reducing its ΔG_H*_ value from the original 0.581 to 0.298 eV. Clearly, doping N atoms can also induce higher HER catalytic activity in the composite Ta_3_N_2_/NG system compared to the pure Ta_3_N_2_/G.

In the composite Mo_3_N_2_/NG system, the T_C3_ and T_C4_ sites can even have near-zero ΔG_H*_ values of −0.005 and 0.083 eV, respectively (Figure 3 and Table S8), indicating significantly high HER catalytic activity. The introduction of N atoms results in higher HER activity compared to the two initial T_C_ sites (0.231 and 0.330 eV). The remaining sites still exhibit poor or relatively weak catalytic activity for HER. Obviously, doping N atoms can endow the composite Mo_3_N_2_/NG system with higher HER catalytic activity. As for the analogous W_3_N_2_/NG system, the introduction of N atoms can bring the HER catalytic activity comparable to the undoped W_3_N_2_/G system. Specifically, all four adsorption sites, including T_C2_ (0.163), T_C3_ (−0.204), T_C4_ (−0.232), and T_C5_ (0.011 eV), can still exhibit high HER catalytic activity due to their small ΔG_H*_ values. The remaining two sites, T_C1_ (0.774) and T_N_ (0.559 eV), show inert or relatively weak HER activity.

Obviously, doping N atoms into graphene can usually induce higher HER catalytic activity of a series of 2D composite TM_3_N_2_/NG systems. The results from AIMD simulations reveal that all four nanostructures can maintain their structural integrity after heating to 500 K for 5 *ps* with a time step of 1 *fs* (Figure S7), suggesting high thermal stability. Additionally, the calculated DOS results show that all the N-doped TM_3_N_2_/NG systems can exhibit typical metallic behavior (Figure S8), primarily determined by the relevant states from TM atoms across the Fermi level.

Coupled with their metallic conductivity and high stability, all four N-doped TM_3_N_2_/NG systems can serve as promising alternative catalysts for HER. Clearly, doping N atoms into graphene can be regarded as an effective strategy for enhancing the HER catalytic activity of 2D composite MXene-based nanostructures.

### 2.3. Structures and HER Catalytic Performance of New 2D Composite MXenes/Si and MXenes/PSi Systems Formed by Coupling the TM_3_N_2_ (TM = Nb, Ta, Mo or W) Monolayer with the Pristine or P-Doped Silicone

#### 2.3.1. Structures and HER Catalytic Activity of 2D Composite TM_3_N_2_/Si Systems

To effectively enhance the HER catalytic activity of TM_3_N_2_ (TM = Nb, Ta, Mo or W) systems, we propose another new strategy through covering 2D silicene onto the four MXene systems. For convenience, we refer to these 2D composite systems as TM_3_N_2_/Si. Unlike the planar graphene, silicene features a 2D wrinkled honeycomb structure due to the weaker ability of Si atoms to form π-bonds compared to carbon atoms in the same group. This unique structural characteristic will facilitate the interaction between the Si atoms in silicene and H* through adopting *sp*^3^ hybridization during the HER reaction on the material’s surface. Consequently, we highly anticipate that these newly formed 2D composite TM_3_N_2_/Si systems will demonstrate high HER catalytic performance, even outperforming their graphene-based counterparts (TM_3_N_2_/G systems).

**Figure 4:**
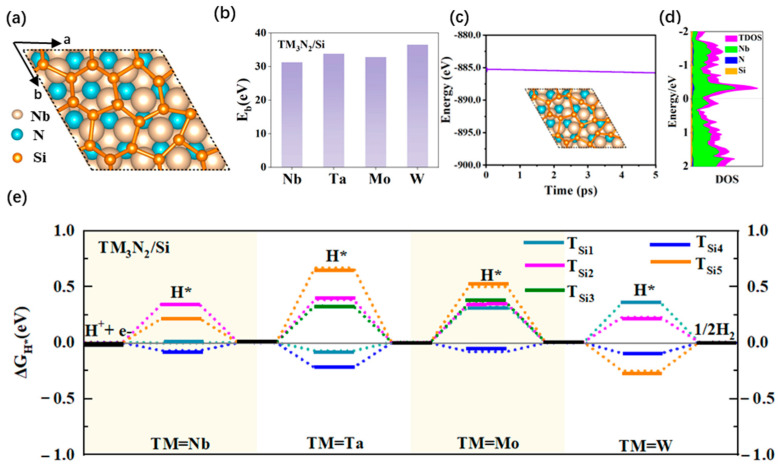
(**a**) Top views of the optimized structure for the Nb_3_N_2_/Si nanostructure. (**b**) The binding energies of TM_3_N_2_/Si (TM = Nb, Ta, Mo, and W) systems. (**c**) The AIMD simulation for Nb_3_N_2_/Si nanostructure at 500 K. Insets: snapshot of the structure after 5 *ps*. (**d**) The DOS of the Nb_3_N_2_/Si nanostructure, where the Fermi level is set to zero. (**e**) The ΔG_H*_ values of different adsorption sites (refer to Figure S9) on the surface of TM_3_N_2_/Si systems. The data correspond to conditions of 1 bar of H_2_ at 298 K.

The optimized structures of the four composite TM_3_N_2_/Si (TM = Nb, Ta, Mo, and W) systems are illustrated in Figure 4a and Figure S9. The computed lattice parameters a/b for these TM_3_N_2_/Si systems fall within the range of 11.028–11.835 Å for TM = Nb, Ta, Mo, and W, respectively (Table S9). Furthermore, their calculated binding energy E_b_ values can be as large as 31.232–36.532 eV per supercell (Figure 4b and Table S9), indicating that depositing 2D silicene onto these four MXene monolayers is energetically favorable, and the resulting composite nanostructures can exhibit considerably high structural stability.

Subsequently, we investigated the HER catalytic activity of the four composite TM_3_N_2_/Si (TM = Nb, Ta, Mo, and W) systems by calculating ΔG_H*_ values of five representative top sites over the Si atoms in the subunit silicene. Specifically, they include the Si atoms over the TM atom, the three-membered ring composed of TM atoms with the corresponding N atom positioned beneath the center of the ring, the three-membered ring composed of TM atoms without the N atom beneath the center, the TM-TM bond, and the TM-N bond, which are denoted T_Si1_, T_Si2_, T_Si3_, T_Si4_ and T_Si5_, respectively (Figure S9). Our computed results reveal that covering 2D silicene can effectively enhance the HER performance of these four MXene monolayers, endowing the composite TM_3_N_2_/Si systems with significant HER catalytic activity (Figure 4e, Tables S8 and S11), even surpassing that of the parallel TM_3_N_2_/G systems.

Specifically, for the Nb_3_N_2_/Si structure, the calculated ΔG_H*_ values for the T_Si1_, T_Si4_ and T_Si5_ sites are as small as −0.006, −0.072 and 0.212 eV, respectively, indicating considerably high HER catalytic activity (Figure 4e, Tables S8 and S11). Especially, the T_Si1_ and T_Si4_ sites can even have a near-zero ΔG_H*_ value, indicating significantly high HER catalytic activity. Additionally, the remaining T_Si2_ (0.340 eV) site can also exhibit good HER activity due to its relatively small ΔG_H*_ value. Clearly, covering the 2D silicene can bring the high HER catalytic activity in the composite Nb_3_N_2_/Si system, even higher than the parallel Nb_3_N_2_/G system, primarily due to the presence of more active sites.

Similarly, depositing the 2D silicene can also endow the analogous Ta_3_N_2_/Si nanostructure with high HER catalytic activity, even surpassing that of the parallel Ta_3_N_2_/G system. To be specific, the T_Si1_ and T_Si4_ sites exhibit small ΔG_H*_ values of −0.077 and −0.227 eV, respectively (Figure 4e, Tables S8 and S11), suggesting significantly high HER catalytic activity of these two sites. Additionally, the T_Si2_ (0.386) and T_Si3_ (0.328 eV) sites also display relatively small ΔG_H*_ values, implying good HER activity. Conversely, the remaining T_Si5_ (0.669 eV) site shows relatively weak HER activity due to its relatively large ΔG_H*_ value.

For the composite Mo_3_N_2_/Si system, the calculated ΔG_H*_ value at the T_Si4_ (−0.081 eV) site can be close to zero (Figure 4e, Tables S8 and S11), suggesting outstanding HER catalytic activity and identifying it as the most active site. Additionally, other sites, including T_Si1_ (0.327), T_Si2_ (0.349), and T_Si3_ (0.351 eV), also exhibit good HER catalytic activity due to their relatively small ΔG_H*_ values. In contrast, the remaining T_Si5_ site shows relatively weak HER activity, as evidenced by its relatively large ΔG_H*_ value of 0.500 eV. Obviously, in contrast to the parallel Mo_3_N_2_/G system, the composite Mo_3_N_2_/Si system can possess higher catalytic activity due to the presence of more active sites. For the analogous W_3_N_2_/Si nanostructure, the T_Si4_ (−0.097 eV) site, with a near-zero ΔG_H*_ value, can serve as the most active site and demonstrate significantly high HER catalytic activity (Figure 4e, Tables S8 and S11). Additionally, the calculated ΔG_H*_ value for the T_Si2_ site is as small as 0.226 eV, suggesting high HER catalytic activity. The remaining two sites, T_Si1_ (0.357) and T_Si5_ (−0.257 eV), can also exhibit good HER activity. Clearly, covering 2D silicene can also endow the composite W_3_N_2_/Si system with high HER catalytic activity, although it shows relatively lower catalytic activity compared to the parallel W_3_N_2_/G system, which has more active sites.

In addition, AIMD simulations were conducted at 500 K to evaluate the thermal stability of the 2D TM_3_N_2_/Si (TM = Nb, Ta, Mo, and W) systems. As shown in Figure 4c and Figure S10, no significant structural deformation can be observed after 5 *ps*, indicating their high thermal stability. Furthermore, the computed DOS results reveal that all four composite TM_3_N_2_/Si systems display metallic behavior, which can be mainly dominated by the relevant states from TM atoms across the Fermi level, as illustrated in Figure 4d and Figure S11.

Obviously, in addition to their high stability and metallic conductivity, all four new 2D TM_3_N_2_/Si systems can uniformly exhibit high HER catalytic activity, with the relevant Si atoms on the subunit silicene serving as the active sites. Particularly, the TM_3_N_2_/Si (TM = Nb, Ta and Mo) systems demonstrate even higher HER activity compared to their parallel TM_3_N_2_/G systems. Covering the related 2D MXene monolayers with silicene can be considered another new strategy for developing low-cost and high-performance HER electrocatalysts.

#### 2.3.2. Structures and HER Catalytic Activity of 2D Composite TM_3_N_2_/PSi Systems

Based on the discussions above, high HER catalytic activity can be observed in the 2D composite TM_3_N_2_/Si (TM = Nb, Ta, Mo, and W) systems. However, it is also anticipated that the HER catalytic activity of these composite systems can be further enhanced by minimizing the ΔG_H*_ values of relevant sites to be closer to zero. In this work, an effective strategy is proposed to optimize the adsorption state of H* on TM_3_N_2_/Si through doping P atom with an atomic radius similar to Si atom, but possessing larger electronegativity.

Initially, the 2D composite TM_3_N_2_/PSi (TM = Nb, Ta, Mo, and W) nanostructures are constructed by coating the P-doped silicene onto the TM_3_N_2_ monolayers (Figure S13), which can be considered the doped TM_3_N_2_/Si systems with P atoms incorporated into the Si sites of the silicene. The calculated lattice parameters for these doped TM_3_N_2_/PSi systems are in the range of 11.037–11.869 Å (Table S9), all of which can be close to those of the corresponding pristine TM_3_N_2_/Si systems (11.028–11.835 Å), suggesting the negligible effect of P-doping on the entire structures. Similar to the pristine TM_3_N_2_/Si systems (31.232–36.532 eV), the doped TM_3_N_2_/PSi systems can also exhibit substantial binding energy E_b_ values of 31.472–35.914 eV per supercell (Figure S12 and Table S9), indicating their high structural stability.

**Figure 5:**
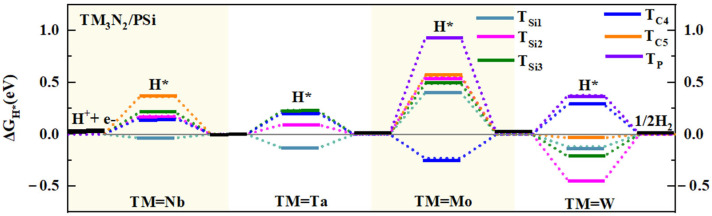
ΔG_H*_ values of different adsorption sites (refer to Figure S13) on the surface of the TM_3_N_2_/PSi (TM = Nb, Ta, Mo, and W) systems. The data correspond to conditions of 1 bar of H_2_ at 298 K.

Subsequently, we investigated the impact of P-doping on the HER catalytic performance of 2D composite TM_3_N_2_/Si (TM = Nb, Ta, Mo, and W) systems by considering all five representative Si sites (T_Si1_–T_Si5_) and the doped P site (T_P_), as depicted in Figure S13. Specifically, when doping P atoms into the subunit silicene in Nb_3_N_2_/Si, the T_Si1_ site (−0.036 eV) can demonstrate a near-zero ΔG_H*_ value, which can still serve as the most active site (Figure 5 and Table S11). Additionally, the HER catalytic activity of T_Si2_ site can be effectively enhanced by reducing its ΔG_H*_ value from the original 0.340 to 0.174 eV (Figure 5 and Table S11). The calculated ΔG_H*_ values of the T_Si3_ and the T_Si4_ sites are 0.212 eV and 0.154 eV, respectively, indicating their notable HER catalytic activity. In addition, the remaining T_Si5_ site (0.360 eV) also exhibits certain HER activity. Obviously, doping P atoms can effectively boost the HER catalytic activity of Nb_3_N_2_/Si, and higher HER activity can be observed in the composite Nb_3_N_2_/PSi system due to the presence of more active sites.

A similar situation can be observed in the composite Ta_3_N_2_/PSi system. Specifically, the HER catalytic activity of the T_Si2_ site is significantly enhanced by reducing the ΔG_H*_ value from 0.386 to 0.093 eV (Figure 5 and Table S11). In view of a near-zero ΔG_H*_ value, the T_Si2_ site can be used as the most active site on Ta_3_N_2_/PSi. Doping P atoms can effectively alter the ΔG_H*_ values of the T_Si3_ and T_Si4_ sites from the original 0.328 to 0.231 eV and −0.227 eV to 0.207 eV, respectively, indicating enhanced HER catalytic activities at these two sites. Additionally, the T_Si1_ site (−0.135 eV) also exhibits considerably high HER activity due to its small ΔG_H*_ value. Obviously, doping P atoms can lead to higher HER catalytic activity in the composite Ta_3_N_2_/PSi system as well.

In the case of the composite Mo_3_N_2_/PSi system, although the doping of P atoms may introduce a somewhat negative effect, good HER catalytic activity can still be observed. As illustrated in Figure 5 and Table S11, the calculated ΔG_H*_ value for the T_Si4_ site is as small as −0.232 eV, implying a considerably high HER catalytic activity and making it the most active site on Mo_3_N_2_/PSi. In contrast, the remaining sites still demonstrate relatively weak catalytic activity for HER. As for the analogous W_3_N_2_/PSi system, doping P atoms can effectively decrease the ΔG_H*_ values of the T_Si1_ and T_Si5_ sites from the original 0.357 to −0.120 eV and −0.257 eV to −0.035 eV, respectively, signifying an enhancement in their HER catalytic activities (Figure 5 and Table S11). The T_Si3_ site (−0.213 eV) can also exhibit considerably high HER activity due to its small ΔG_H*_ value. Additionally, the T_Si4_ site (0.289 eV) can present good HER activity, while the remaining T_Si2_ (−0.446 eV) and T_P_ (0.375 eV) sites can display certain HER activity. Therefore, compared to the undoped W_3_N_2_/Si system, the introduction of P can lead to slightly higher HER catalytic activity.

Clearly, all four P-doped TM_3_N_2_/PSi monolayers (TM = Nb, Ta, Mo, and W) can exhibit high HER catalytic activity, with Nb_3_N_2_/PSi and Ta_3_N_2_/PSi systems exhibiting much higher HER activity than their counterparts with undoped silicene. The AIMD simulations show that all four nanostructures can maintain their structural integrity after heating at 500 K for 5 *ps* with a time step of 1 *fs* (Figure S14), confirming their high thermal stability. In addition, the computed DOS results reveal that all the P-doped TM_3_N_2_/PSi systems can display metallic behavior uniformly (Figure S15), which are mainly dominated by the relevant states from TM atoms across the Fermi level. Combining their metallic conductivity and high stability, all four P-doped TM_3_N_2_/PSi systems can hold promise as viable alternative catalysts for HER.

### 2.4. HER Catalytic Mechanisms

**Figure 6:**
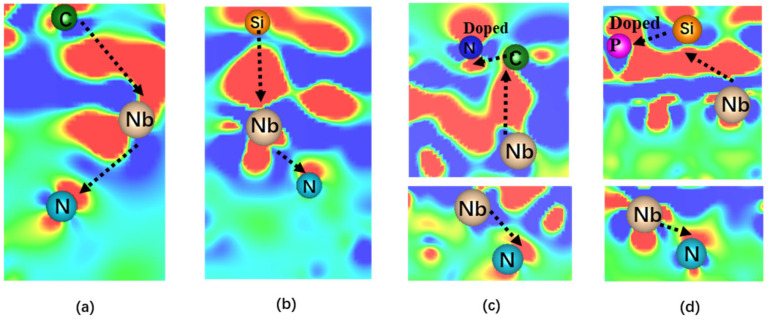
The charge density difference (Δρ) of Nb_3_N_2_/G (**a**), Nb_3_N_2_/Si (**b**), Nb_3_N_2_/NG (**c**) and Nb_3_N_2_/PSi (**d**) systems, where the red and blue colors represent gaining and losing electrons, respectively, and the relevant electron transfer processes are displayed.

From the findings above, it is evident that the deposition of graphene and silicene on the four 2D MXene systems (TM_3_N_2_, where TM = Nb, Ta, Mo, and W) can lead to high HER catalytic activity. Furthermore, doping with N or P atoms can induce higher HER catalytic performance in the doped TM_3_N_2_/NG (TM = Nb, Ta and Mo) and TM_3_N_2_/PSi (TM = Nb and Ta) systems. To understand the underlying reasons for their high HER catalytic activity, we conducted the charge density difference (Δρ) calculations on these systems, as illustrated in Figure 6 and Figure S16.

Initially, our focus was on the four 2D composite TM_3_N_2_/G (TM = Nb, Ta, Mo, and W) systems. As depicted in Figure 6a and Figure S16, upon coating 2D TM_3_N_2_ with graphene, a complex electron transfer process (referred to as C → TM → N) can be observed in TM_3_N_2_/G, where the C atoms serve as electron donors, while the N atoms act as electron acceptors. This electron transfer process can effectively activate the relevant C atoms by adjusting the electron density, leading to superior HER catalytic activity on the subunit graphene in TM_3_N_2_/G.

In order to explore the reason behind the effective improvement in the HER catalytic activity of the 2D composite TM_3_N_2_/G systems after N-doping, we performed Δρ calculations by choosing three composite TM_3_N_2_/NG (TM = Nb, Ta and Mo) systems, which displayed significantly enhanced HER activity in the carbon atoms adjacent to the doped N atoms. As shown in Figure 6c and Figure S16, doping N atoms (denoted N_d_) into graphene can induce a more complex electron transfer process (N ← TM → C → N_d_) in the TM_3_N_2_/NG, where the relevant C atoms neighboring to the doped N atoms can play a dual role, serving as both electron donors and electron acceptors. This enables a more effective modulation of the electron density on the relevant carbon atoms, resulting in higher HER activity.

Similarly, when covering silicene onto the TM_3_N_2_ structures (TM = Nb, Ta, Mo, and W), a complex electron transfer process (i.e., Si → TM → N) can be observed in the composite TM_3_N_2_/Si systems. In this process, the Si atoms act as electron donors, while the N atoms serve as electron acceptors (Figure 6b and Figure S16). This can make the relevant Si atoms highly active sites for HER by effectively modulating their electron density. Furthermore, when doping P atoms into the silicene, the enhanced HER catalytic activity can be observed in the 2D composite TM_3_N_2_/PSi (TM = Nb and Ta) system. This can be mainly attributed to the case that a more complex electron transfer process (i.e., N ← TM → Si → P) can take place in the doped TM_3_N_2_/PSi (TM = Nb and Ta) systems, where the related Si atoms adjacent to the dopant P can assume dual roles as both electron donors and electron acceptors (Figure 6d and Figure S16). This can allow for finer tuning of the electron density at the relevant Si atoms, leading to increased HER catalytic activity. Overall, the occurrence of complex electron transfer process can be responsible for the effective enhancement of HER catalytic performance in these composite MXene-based systems.

## 3. Computational Methods

The density functional theory (DFT) calculations within the framework of VASP [66,67] were performed through the generalized gradient approximation (GGA) with the Perdew–Burke–Ernzerhof (PBE) exchange-correlation functional [68]. The projector-augmented plane wave (PAW) was adopted to describe the interaction between electrons and ions [69,70], where the semi-empirical van der Waals (vdW) correction was included to account for the dispersion interaction [71]. Geometric optimization of pristine TM_3_N_2_ (TM = Nb, Ta, Mo, and W) monolayers with a small unit cell was conducted using a 9 × 9 × 1 Monkhorst–Pack grid of *k*-points. For the structural optimization of composite systems with larger supercells, 3 × 3 × 1 *k*-points were used. Additionally, all calculations related to hydrogen evolution were performed using 3 × 3 × 1 *k*-points, while the density of states (DOSs) was calculated by adopting 63 *k*-points. The truncation energy of the plane wave basis set was 400 eV. To avoid interactions between images in the repeated supercell, vacuum spaces wider than 20 Å were employed along the non-periodic direction. For all the calculations, the energy convergence threshold was 10^−4^ eV, and all forces on each atom were below 0.05 eV/Å.

The structural stability of TM_3_N_2_ MXene systems was assessed by calculating the cohesive energy via the following formula:

E_coh_ = (mE_N_ + nE_TM_ − E_TM3N2_)/(m + n) (1)

where E_TM_, E_N_ and E_TM3N2_ are the total energies of a single TM atom, a single N atom, and the TM_3_N_2_ monolayers; m and n are the number of N and TM atoms in the supercell, respectively.

Furthermore, the binding energy (E_b_) of the relevant composite structures can be calculated using the following formula:

E_b_ = E_MXenes_ + E_X_ − E_MXenes-X_ (2)

where E_MXenes-X_ is the total energy of the composite structure, E_MXenes_ is the energy of 2D MXenes, and E_X_ is the energy of the pure or doped graphene/silicene monolayer. It is noteworthy that a positive E_b_ value indicates a formation process with favorable energy. The dynamic stability of the structure is evaluated through phonon calculations, which employ the finite displacement method as implemented in the Phonopy code [72]. Thermal stability is assessed through ab initio molecular dynamics (AIMD) simulations [73]. By implementing the Nosé–Hoover method, the simulations are performed in the NVT ensemble at a constant temperature of 500 K [74]. For the studied systems, the total simulation duration is set to 5 *ps*, with a time step of 1 *fs*. In addition, the electron localization function (ELF) is used to analyze the bonding characteristics of the studied systems [75,76]. It is known that the ELF can be characterized in the form of a contour plot within a range of 0–1. The regions close to 1 indicate areas of high electron density; the regions around 0.5 mean a homogeneous electron gas; and the regions near 0 suggest areas of low electron density.

The Gibbs free energy of H* adsorption can be calculated by the following formula:

ΔG_H*_ = ΔE_H*_+ ΔE_ZPE_ − TΔS (3)

where ΔE_H*_, ΔE_ZPE_ and ΔS are the hydrogen chemisorption energy, the difference in zero-point energy between the absorbed and the gas phase, and the entropy change of H* adsorption, respectively. In this work, TΔS and ΔE_ZPE_ were obtained by the scheme proposed by Nørskov et al. [77]. Specifically, ΔS was calculated by the equation ΔS = S(H*) − 1/2S(H_2_) ≈ −1/2S(H_2_), due to the negligible vibrational entropy of H*. Considering that TS(H_2_) is 0.410 eV for H_2_ at 298 K and 1 atm [78], the corresponding TΔS can be −0.205 eV. In addition, the equation ΔE_ZPE_ = E_ZPE(H*)_ − 1/2E_ZPE(H2)_ was employed to assess ΔE_ZPE_ for H*. It is noteworthy that our calculated E_ZPE(H2)_ value is about 0.298 eV, which is close to the reported result by Nørskov et al. [77]. In addition, we performed the related convergence tests concerning the choice of *k*-points and truncation energy by sampling the Nb_3_N_2_ supercell structure. The computational settings compared included *k*-points of 3 × 3 × 1 versus 5 × 5 × 1, along with truncation energies of 400 eV, 450 eV, and 500 eV. The results demonstrate that the obtained lattice constants, bond lengths, and ΔG_H*_ values can remain consistent across these different settings (Table S12). This indicates that the *k*-points of 3 × 3 × 1 and the truncation energy of 400 eV used in this study can provide reliable calculation results.

## 4. Conclusions

Through the detailed DFT calculations, we designed a series of novel 2D composite nanomaterials based on the excellent TM_3_N_2_ MXenes (TM = Nb, Ta, Mo, and W) and investigated their stability and HER catalytic activity. The following intriguing findings were achieved.

(1) Covering 2D graphene can effectively improve the HER catalytic activity of the four TM_3_N_2_ MXene systems. The resulting composite TM_3_N_2_/G structures can uniformly exhibit high stability, metallic conductivity, and considerably high HER catalytic activity (much higher than the standalone TM_3_N_2_ systems). The relevant carbon atoms in the subunit graphene can serve as active sites for HER.
(2) The TM_3_N_2_ systems coated with the N-doped graphene can also demonstrate high stability, metallic conductivity, and significantly high HER catalytic activity. Particularly, doping N atoms into the subunit graphene can result in higher HER catalytic activity in the composite TM_3_N_2_/G systems (TM = Nb, Ta and Mo), with the HER activity of relevant carbon atoms adjacent to the nitrogen dopant being effectively boosted.
(3) The coating of 2D buckled silicene can significantly enhance the HER catalytic activity of the four TM_3_N_2_ MXene systems. The resulting composite TM_3_N_2_/Si structures can display high stability, metallic conductivity, and remarkable HER catalytic performance. The relevant silicon atoms in the subunit silicene can act as active sites for HER. Notably, all three TM_3_N_2_/Si (TM = Nb, Ta and Mo) systems outperform their TM_3_N_2_/G counterparts in HER catalytic activity, indicating the superior effectiveness of silicene coupling.
(4) The composite TM_3_N_2_/PSi, formed by coating the P-doped silicene, can exhibit high stability, metallic conductivity, and high HER catalytic activity. Especially, the incorporation of P atoms into silicene can endow Nb_3_N_2_/PSi and Ta_3_N_2_/PSi systems with enhanced HER activity in comparison to those covered with undoped silicene, enhancing the HER activity of silicon atoms adjacent to the phosphorus dopant.

Overall, we propose several new strategies to effectively enhance the HER catalytic performance of the four 2D layered TM_3_N_2_ MXenes (TM = Nb, Ta, Mo, and W) by coupling them with pristine or N/P-doped graphene and silicene. The relevant catalytic mechanisms were analyzed in detail. Combined with suitable H* adsorption states, metallic conductivity, and high stability, these composite systems based on TM_3_N_2_ MXenes can serve as non-precious and highly efficient HER electrocatalysts. This work can provide valuable theoretical insights for designing new HER catalysts based on excellent MXenes.

## Data Availability

The original contributions presented in this study are included in the article. Further inquiries can be directed to the corresponding authors.

## Supplementary Materials

The following supporting information can be downloaded at https://www.mdpi.com/article/10.3390/molecules30112401/s1. Figure S1: Top and side views of the optimized structures (a–c), corresponding phonon spectra (d–f), ELF maps (g–i), and DOSs (j–l) for the Ta_3_N_2_, Mo_3_N_2_ and W_3_N_2_ monolayers, respectively; Figure S2: (a–d) Top views of the TM_3_N_2_/G (TM = Nb, Ta, Mo, and W) nanostructures and typical obtained adsorption sites of H* on the surface of these composite systems; Figure S3: Variations of total energy for Ta_3_N_2_/G (a), Mo_3_N_2_/G (b), and W_3_N_2_/G (c) at 500 K during AIMD simulations. Insets: snapshots of the structures after 5 *ps*; Figure S4: DOSs of the Ta_3_N_2_/G (a), Mo_3_N_2_/G (b), and W_3_N_2_/G (c) nanostructures; Figure S5: Binding energies of TM_3_N_2_/NG (TM = Nb, Ta, Mo, and W) nanostructures; Figure S6: (a–d) Top views of the TM_3_N_2_/NG (TM = Nb, Ta, Mo, and W) nanostructures and typical obtained adsorption sites of H* on the surface of these composite systems; Figure S7: Variations in total energy for Nb_3_N_2_/NG (a), Ta_3_N_2_/NG (b), Mo_3_N_2_/NG (c), and W_3_N_2_/NG (d) at 500 K during AIMD simulations. Insets: snapshots of the structures after 5 *ps*; Figure S8: DOSs of Nb_3_N_2_/NG (a), Ta_3_N_2_/NG (b), Mo_3_N_2_/NG (c), and W_3_N_2_/NG (d) systems; Figure S9: (a–d) Top views of the TM_3_N_2_/Si (TM = Nb, Ta, Mo, and W) nanostructures, and typical obtained adsorption sites of H* on the surface of these composite systems; Figure S10: Variations in total energy for Ta_3_N_2_/Si (a), Mo_3_N_2_/Si (b), and W_3_N_2_/Si (c) at 500 K during AIMD simulations. Insets: snapshots of the structures after 5 *ps*; Figure S11: DOSs of the Ta_3_N_2_/Si (a), Mo_3_N_2_/Si (b), and W_3_N_2_/Si (c) systems; Figure S12: Binding energies of TM_3_N_2_/PSi (TM = Nb, Ta, Mo, and W) nanostructures; Figure S13: (a–d) Top views of the TM_3_N_2_/PSi (TM = Nb, Ta, Mo, and W) nanostructures and typical obtained adsorption sites of H* on the surface of these composite systems; Figure S14: Variations in total energy for Nb_3_N_2_/PSi (a), Ta_3_N_2_/PSi (b), Mo_3_N_2_/PSi (c), and W_3_N_2_/PSi (d) at 500 K during AIMD simulations. Insets: snapshots of the structures after 5 *ps*; Figure S15: DOSs of the Nb_3_N_2_/PSi (a), Ta_3_N_2_/PSi (b), Mo_3_N_2_/PSi (c), and W_3_N_2_/PSi (d) systems; Figure S16: Charge density difference (△ρ) of Ta_3_N_2_/G (a), Mo_3_N_2_/G (b), W_3_N_2_/G (c), Ta_3_N_2_/Si (d), Mo_3_N_2_/Si (e), W_3_N_2_/Si (f), Ta_3_N_2_/NG (g), Mo_3_N_2_/NG (h), and Ta_3_N_2_/PSi (i) nanostructures. Red and blue represent gaining and losing electrons, respectively, and the relevant electron transfer processes are displayed; Table S1: Lattice constants lengths and E_coh_ of TM_3_N_2_; Table S2: Corresponding bond lengths of TM_3_N_2_ in the experimental synthesis system; Table S3: Calculated Bader charges on N atoms in the TM_3_N_2_ (TM = Nb, Ta, Mo, and W) systems; Table S4: Elastic coefficients for the TM_3_N_2_ monolayers; Table S5: Calculated ΔG_H*_ values at T_TM_, B_Nb-Nb_, H_1_, and H_2_ sites for TM_3_N_2_ systems; Table S6: Computed lattice constants and binding energy (E_b_) for 2D composite MXenes/G and MXenes/NG nanostructures; Table S7: TM-TM, TM-N, C-TM, and C-C bond lengths for 2D composite MXenes/G and MXenes/NG nanostructures; Table S8: Calculated ΔG_H*_ values at the T_C1_–T_C5_ and T_N_ sites for MXenes/G and MXenes/NG systems; Table S9: Computed lattice constants and binding energy (E_b_) for 2D composite MXenes/Si and MXenes/PSi nanostructures; Table S10: Si-TM, P-TM, TM-TM, TM-N, Si-Si, and Si-P bond lengths for 2D composite MXenes/Si and MXenes/PSi nanostructures; Table S11: Calculated ΔG_H*_ values at the T_Si1_–T_Si5_ and T_P_ sites for MXenes/Si and MXenes/PSi systems; Table S12: Calculated lattice constants, bond lengths, and ΔG_H*_ values at the H_1_ and H_2_ sites for the sampled Nb_3_N_2_ supercell structure using the different *k*-points and truncation energies (E_cut_).
