# Supplementary material for: Constructing Novel 2D Composite Nanomaterials by Coupling Graphene or Silicene with TM3N2 MXene (TM = Nb, Ta, Mo, and W) to Achieve Highly Efficient HER Catalysts
Source: Molecules. 2025 May 30;30(11):2401. doi: 10.3390/molecules30112401 (PMC12156383; doi:10.3390/molecules30112401)
Supplement: Supplementary file 1 [file molecules-30-02401-s001.zip › molecules-3566740-supplementary.pdf]

Supporting Information

Constructing Novel 2D Composite Nanomaterials by Coupling Graphene or Silicene with TM_3N_2 MXene (TM = Nb, Ta, Mo, and W) to Achieve Highly Efficient HER Catalysts

Xiuyi Zhang [1], Guangtao Yu [1,*], Wei Zhang [2,3,*], E Yang [1] and Wei Chen [1,2,4,*]

1 Engineering Research Center of Industrial Biocatalysis, Fujian Provincial Key Laboratory of Advanced Materials Oriented Chemical Engineering, Fujian-Taiwan Science and Technology Cooperation Base of Biomedical Materials and Tissue Engineering, College of Chemistry and Materials Science, Fujian Normal University, Fuzhou 350007, China
2 Academy of Carbon Neutrality of Fujian Normal University, Fuzhou 350007, China
3 Fujian Provincial Key Laboratory of Quantum Manipulation and New Energy Materials, College of Physics and Energy, Fujian Normal University, Fuzhou 350117, China
4 Fujian Provincial Key Laboratory of Theoretical and Computational Chemistry, Xiamen University, Xiamen 361005, China
* Correspondence: yugt@fjnu.edu.cn (G.Y.); zhangwei@fjnu.edu.cn (W.Z.); chenwei@fjnu.edu.cn (W.C.)

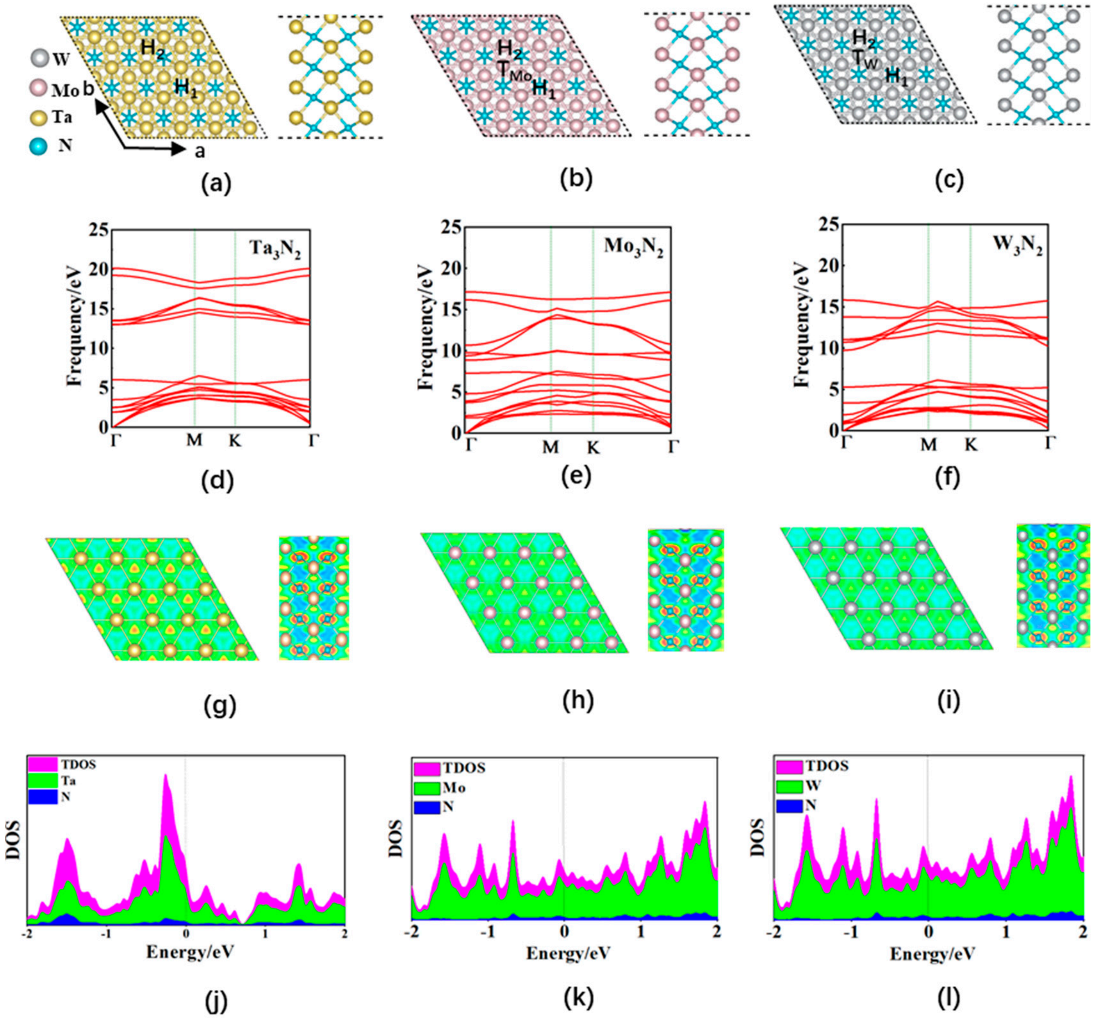

Figure S1. Top and side views of the optimized structures (a–c), corresponding phonon spectra (d–f), ELF maps (g–i), and DOSs (j–l) for the Ta_3N_2, Mo_3N_2 and W_3N_2 monolayers, respectively

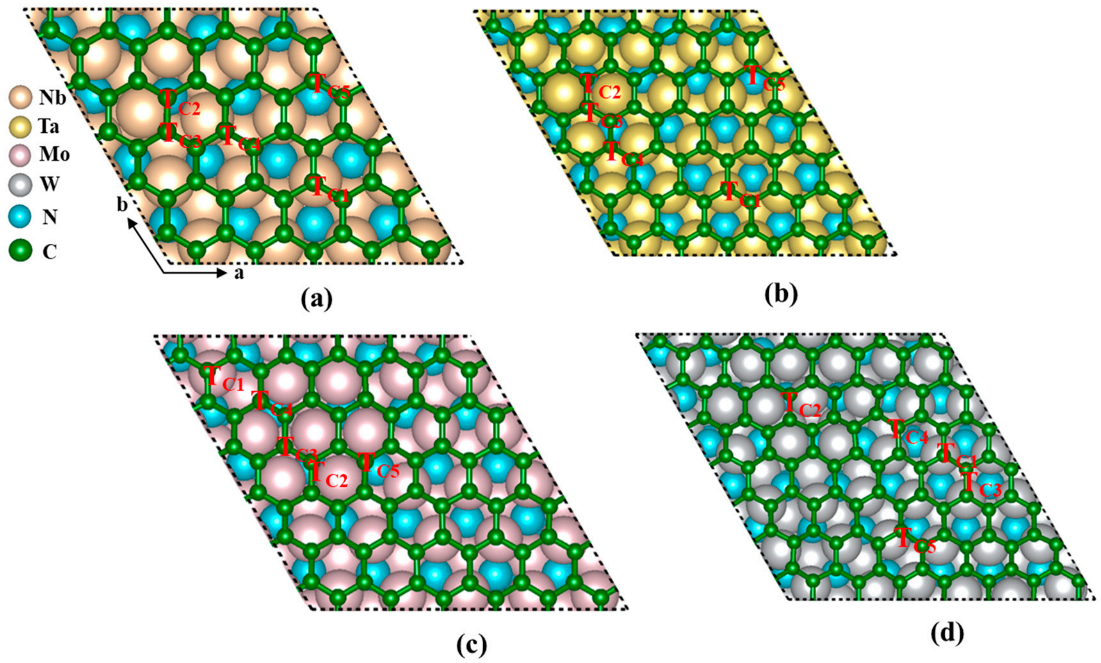

Figure S2. (a–d) Top views of the TM_3N_2/G (TM = Nb, Ta, Mo, and W) nanostructures and typical obtained adsorption sites of H* on the surface of these composite systems

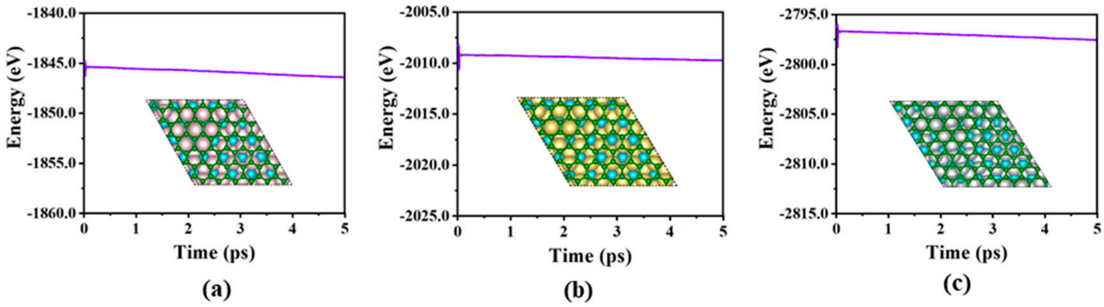

Figure S3. Variations of total energy for Ta_3N_2/G (a), Mo_3N_2/G (b), and W_3N_2/G (c) at 500 K during AIMD simulations. Insets: snapshots of the structures after 5 *ps*

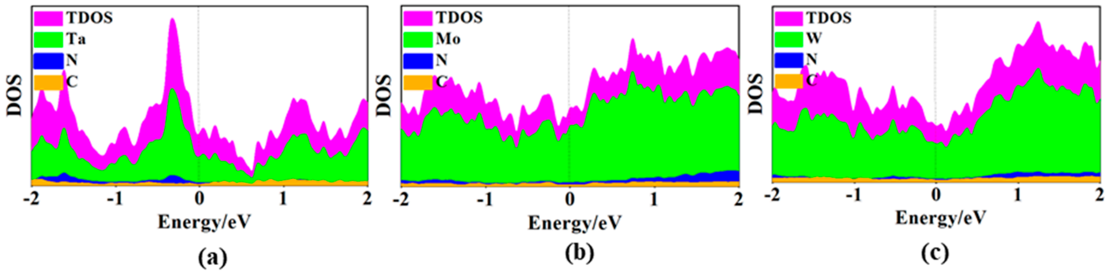

Figure S4. DOSs of the Ta_3N_2/G (a), Mo_3N_2/G (b), and W_3N_2/G (c) nanostructures

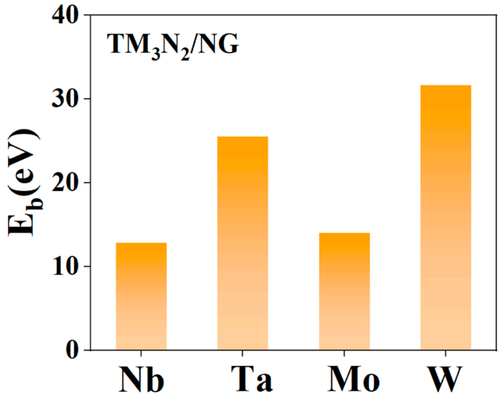

Figure S5. Binding energies of TM_3N_2/NG (TM = Nb, Ta, Mo, and W) nanostructures

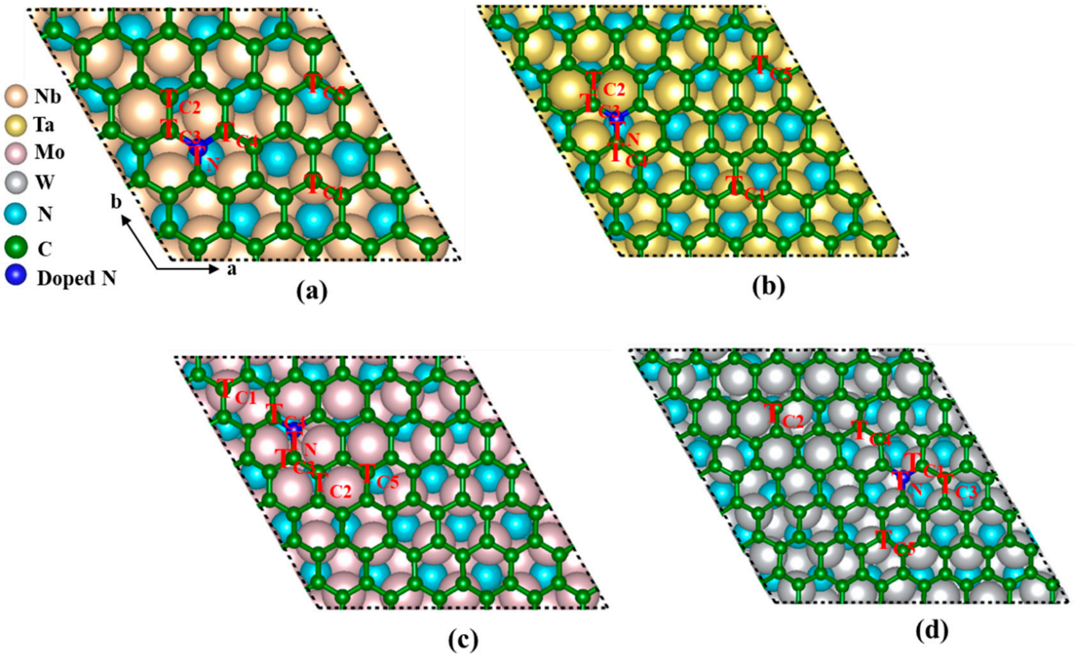

Figure S6. (a–d) Top views of the TM_3N_2/NG (TM = Nb, Ta, Mo, and W) nanostructures and typical obtained adsorption sites of H* on the surface of these composite systems

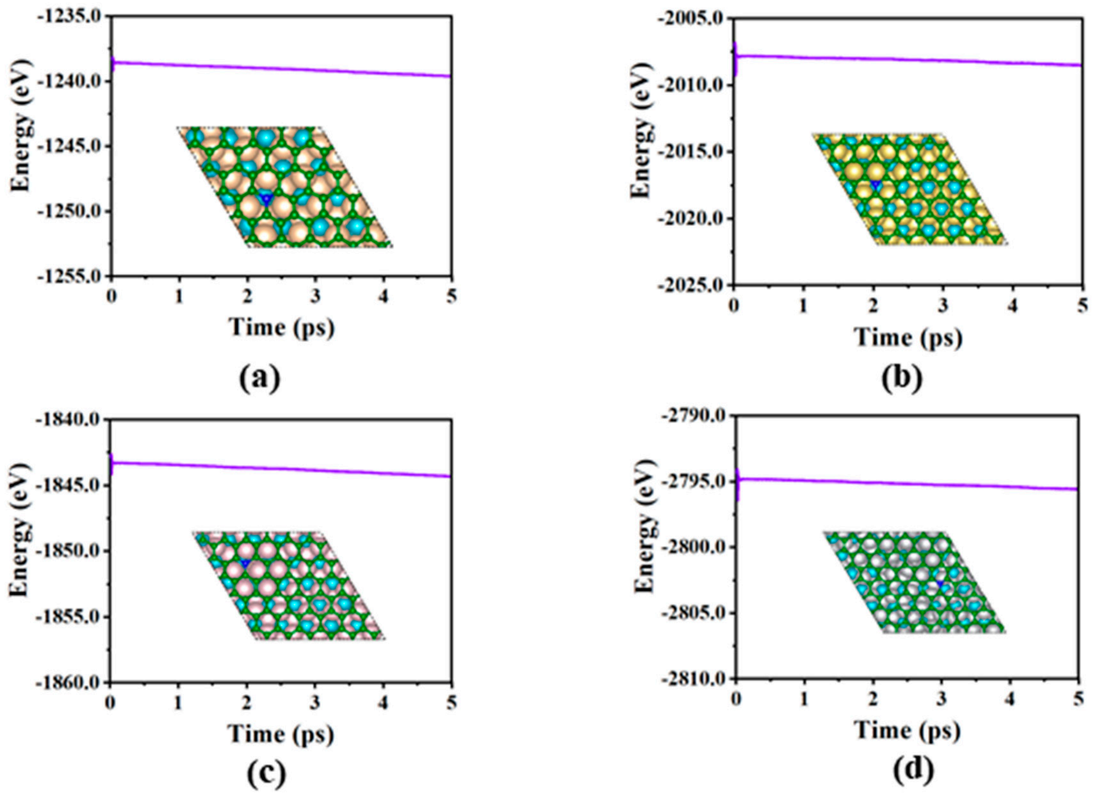

Figure S7. Variations in total energy for Nb_3N_2/NG (a), Ta_3N_2/NG (b), Mo_3N_2/NG (c), and W_3N_2/NG (d) at 500 K during AIMD simulations. Insets: snapshots of the structures after 5 *ps*

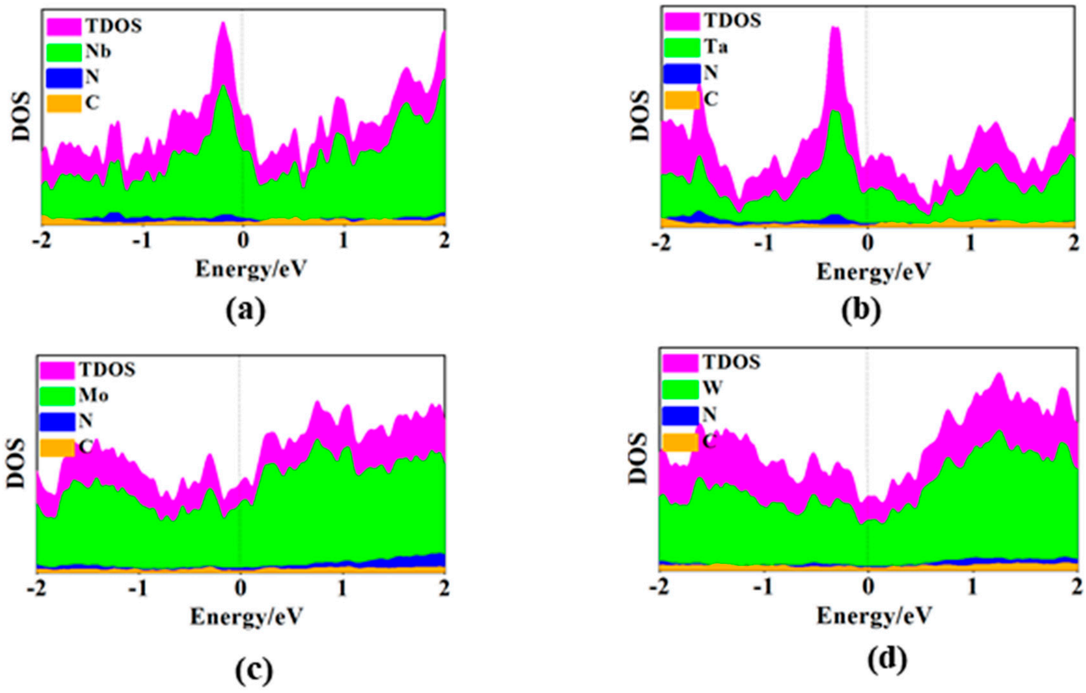

Figure S8. DOSs of Nb_3N_2/NG (a), Ta_3N_2/NG (b), Mo_3N_2/NG (c), and W_3N_2/NG (d) systems

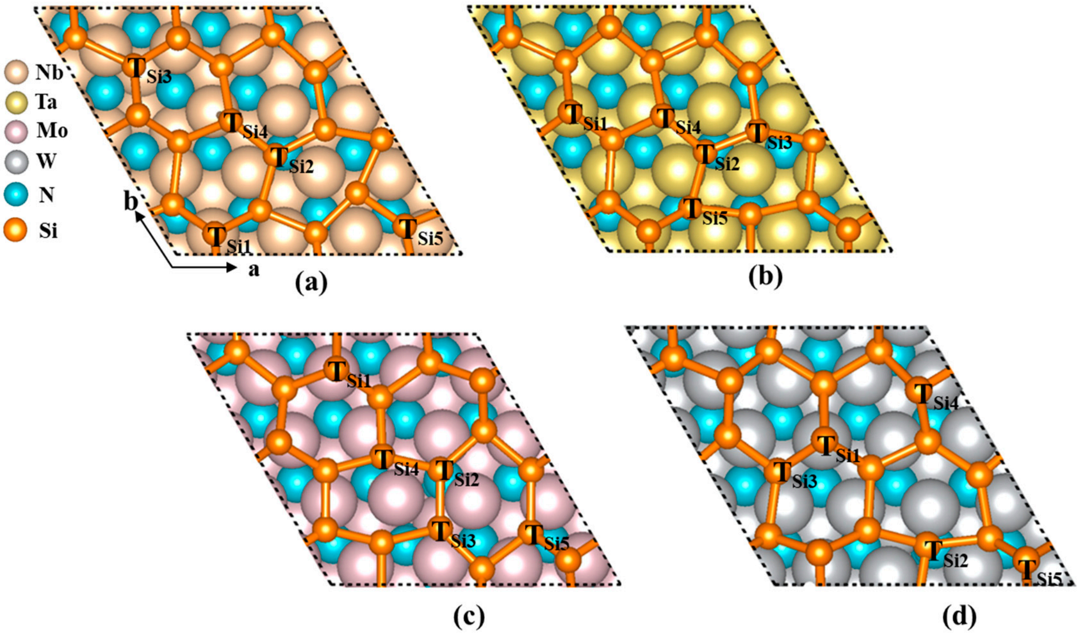

Figure S9. (a–d) Top views of the TM_3N_2/Si (TM = Nb, Ta, Mo, and W) nanostructures, and typical obtained adsorption sites of H* on the surface of these composite systems

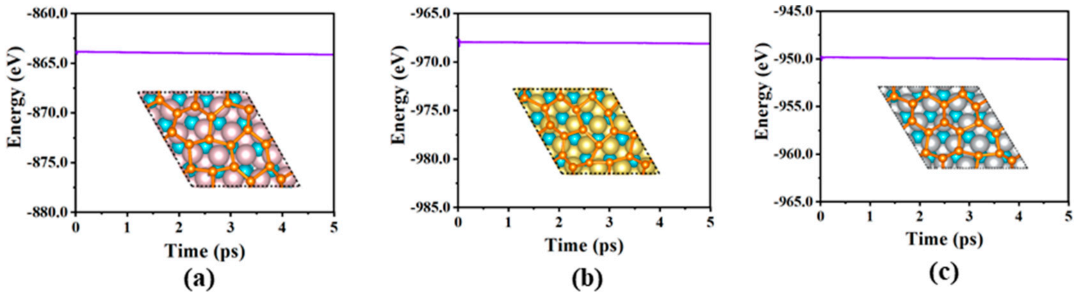

Figure S10. Variations in total energy for Ta_3N_2/Si (a), Mo_3N_2/Si (b), and W_3N_2/Si (c) at 500 K during AIMD simulations. Insets: snapshots of the structures after 5 *ps*

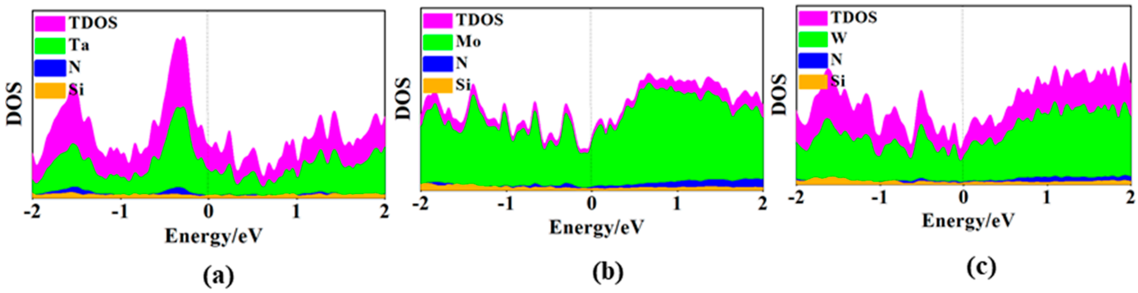

Figure S11. DOSs of the Ta_3N_2/Si (a), Mo_3N_2/Si (b), and W_3N_2/Si (c) systems

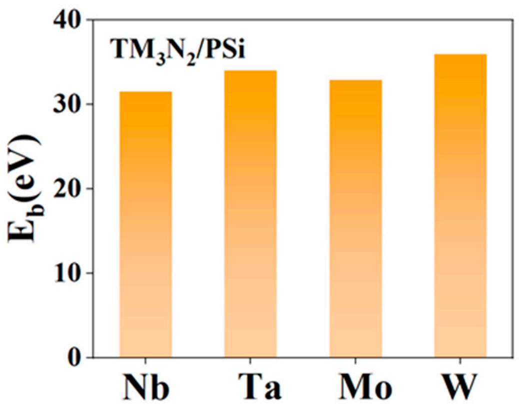

Figure S12. Binding energies of TM_3N_2/PSi (TM = Nb, Ta, Mo, and W) nanostructures

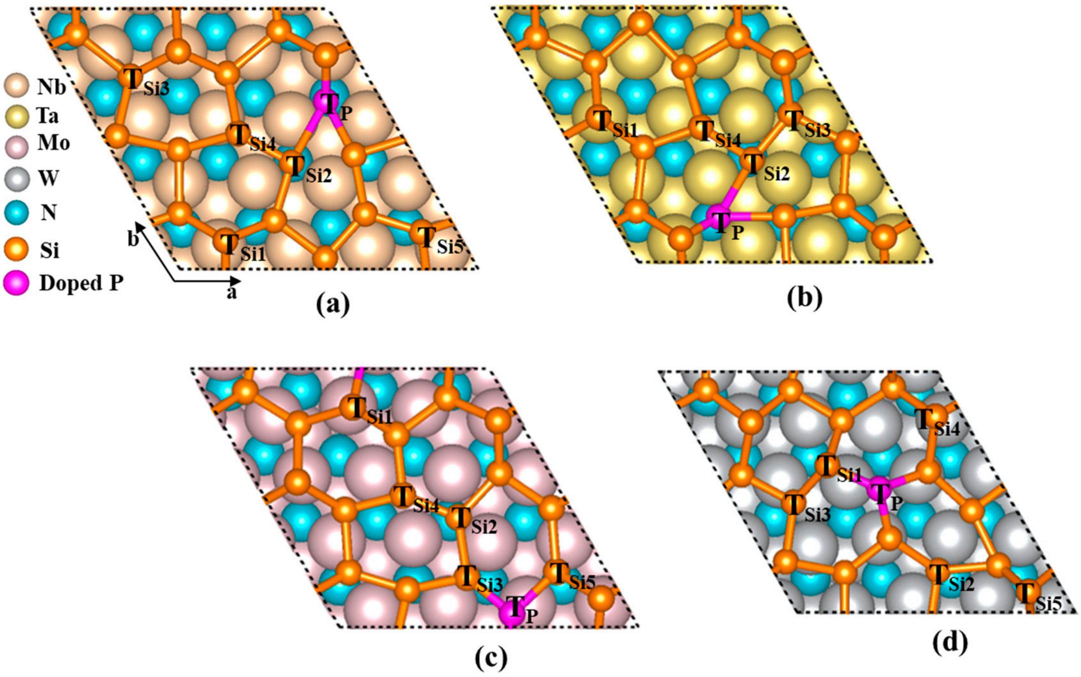

Figure S13. (a–d) Top views of the TM_3N_2/PSi (TM = Nb, Ta, Mo, and W) nanostructures and typical obtained adsorption sites of H* on the surface of these composite systems

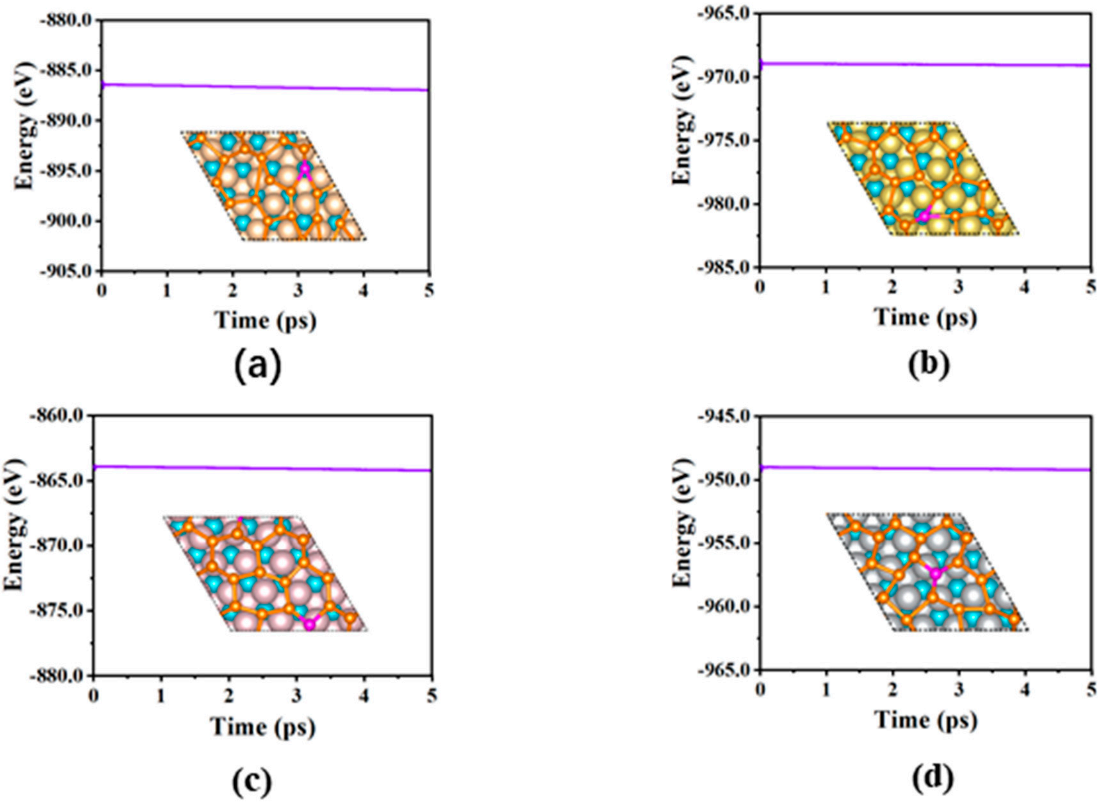

Figure S14. Variations in total energy for Nb_3N_2/PSi (a), Ta_3N_2/PSi (b), Mo_3N_2/PSi (c), and W_3N_2/PSi (d) at 500 K during AIMD simulations. Insets: snapshots of the structures after 5 *ps*

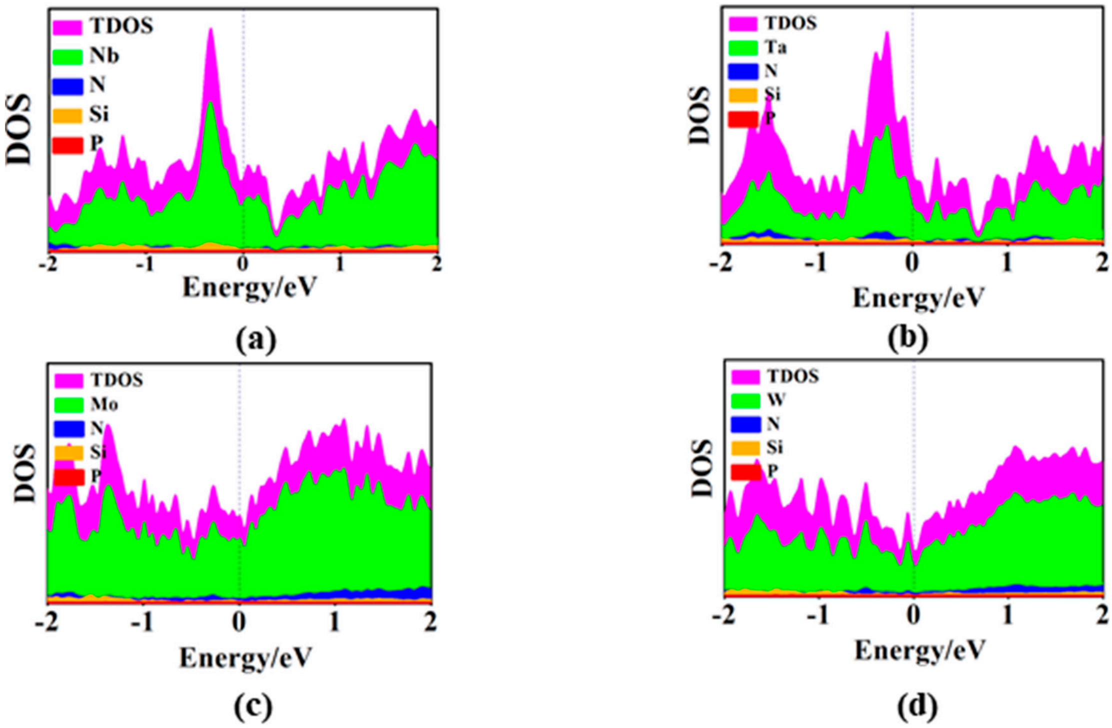

Figure S15. DOSs of the Nb_3N_2/PSi (a), Ta_3N_2/PSi (b), Mo_3N_2/PSi (c), and W_3N_2/PSi (d) systems

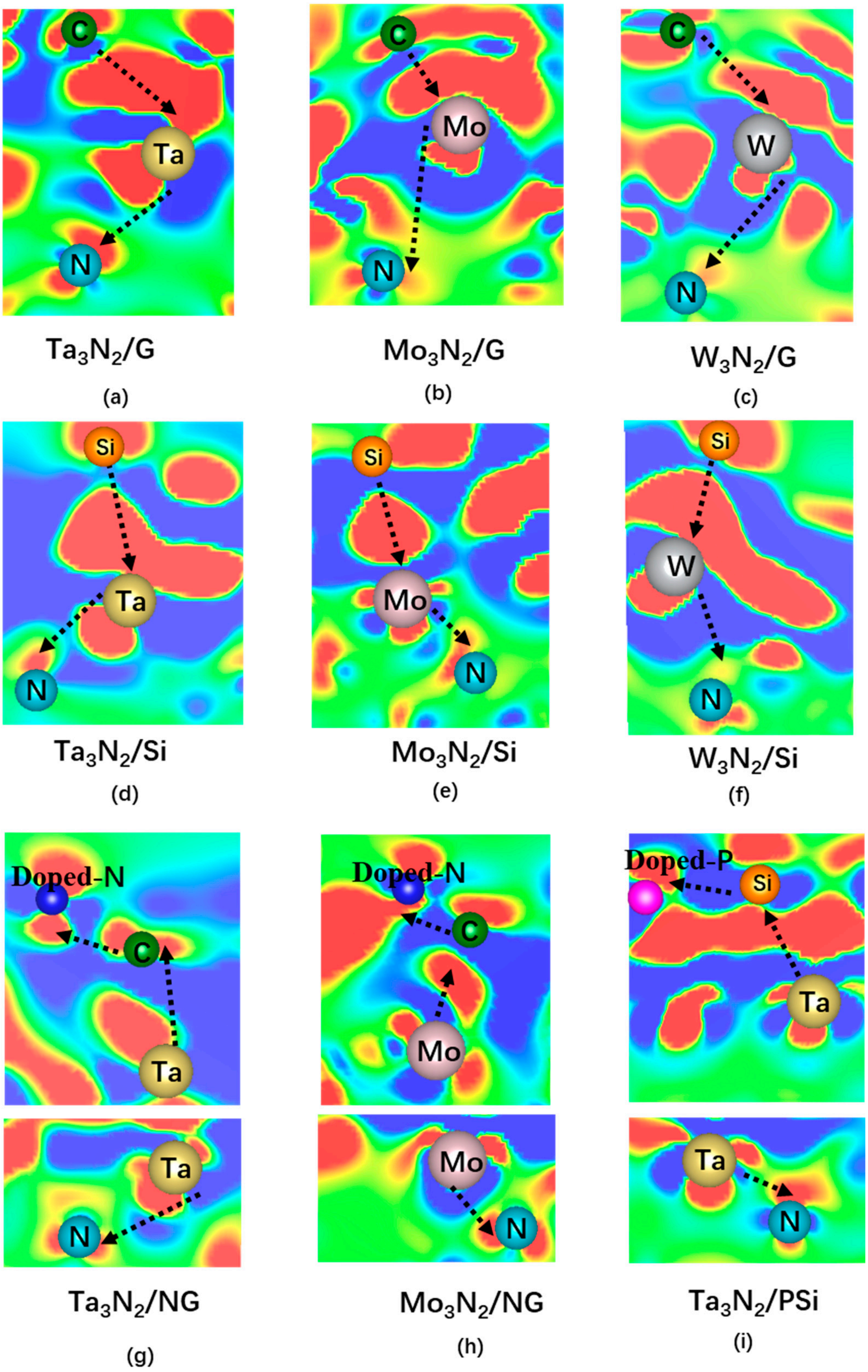

Figure S16. Charge density difference ($\triangle\varrho$) of Ta_3N_2/G (a), Mo_3N_2/G (b), W_3N_2/G (c), Ta_3N_2/Si (d), Mo_3N_2/Si (e), W_3N_2/Si (f), Ta_3N_2/NG (g), Mo_3N_2/NG (h), and Ta_3N_2/PSi (i) nanostructures. Red and blue represent gaining and losing electrons, respectively, and the relevant electron transfer processes are displayed.

Table S1. Lattice constants lengths and E_{coh} of TM_3N_2

Systems	Lattice constants (Å)	E_{coh} (eV)
Nb_3N_2	2.945	6.93
Ta_3N_2	2.882	7.91
Mo_3N_2	2.830	5.78
W_3N_2	2.738	7.50

Table S2. Corresponding bond lengths of TM_3N_2 in the experimental synthesis system

Systems	Bond Lengths (Å)					
	TM-TM			TM-N		
	In this study	In relevant experimental materials		In this study	In relevant experimental materials	
Nb_3N_2	2.945	3.076-3.094	Nb_2N	2.205-2.209	2.172-2.180	Nb_2N
Ta_3N_2	2.882	2.990-3.113	Ta_2N	2.189-2.208	2.203-2.274	Ta_4N_3
Mo_3N_2	2.830	2.829-2.882	Mo_2N	2.147-2.172	2.147	Mo_2N
W_3N_2	2.738	2.740-2.835	W_2N	2.130-2.264	2.023-2.237	W_2N_3

Table S3. Calculated Bader charges on N atoms in the TM_3N_2 (TM = Nb, Ta, Mo, and W) systems

Systems	Charge (|e|)
Nb_3N_2	-1.39
Ta_3N_2	-1.46
Mo_3N_2	-1.25
W_3N_2	-1.44

Table S4. Elastic coefficients for the TM_3N_2 monolayers

Systems	$C_{11} = C_{22}$	C_{12}	C_{66}
Nb_3N_2	1490.892	676.019	407.436
Ta_3N_2	1945.656	815.146	565.255
Mo_3N_2	1537.410	450.198	543.606
W_3N_2	2404.304	894.274	755.016

Table S5. Calculated ΔG_{H^*} values at T_{TM}, $B_{Nb\text{-}Nb}$, H_1, and H_2 sites for TM_3N_2 systems

Systems	Adsorption	ΔG_{H^*} (eV)
Nb_3N_2	T_{Nb}	---
	$B_{Nb\text{-}Nb}$	---
	H_1	-1.016
	H_2	-1.183
Ta_3N_2	T_{Ta}	---
	$B_{Ta\text{-}Ta}$	---
	H_1	-1.115
	H_2	-1.212
Mo_3N_2	T_{Mo}	-0.266
	$B_{Mo\text{-}Mo}$	---
	H_1	-0.622
	H_2	-0.903
W_3N_2	T_W	-0.435
	$B_{W\text{-}W}$	---
	H_1	-0.610
	H_2	-0.898

Table S6. Computed lattice constants and binding energy (E_b) for 2D composite MXenes/G and MXenes/NG nanostructures

Systems	a (Å)	b(Å)	E_b(eV)
Nb_3N_2/G	12.139	12.139	12.344
Ta_3N_2/G	14.621	14.607	25.082
Mo_3N_2/G	14.498	14.498	14.374
W_3N_2/G	16.783	16.718	31.689
Nb_3N_2/NG	12.129	12.129	12.808
Ta_3N_2/NG	14.623	14.614	25.516
Mo_3N_2/NG	14.490	14.490	14.028
W_3N_2/NG	16.777	16.711	31.653

Table S7. TM-TM, TM-N, C-TM, and C-C bond lengths for 2D composite MXenes/G and MXenes/NG nanostructures

Systems	C-TM(Å)	TM-TM(Å)	TM-N(Å)	C-C(Å)	C-N(Å)
Nb_3N_2/G	2.224-2.596	2.799-3.234	2.182-2.366	1.389-1.432	
Ta_3N_2/G	2.199-2.597	2.803-3.186	2.123-2.348	1.384-1.456	
Mo_3N_2/G	2.158-2.575	2.450-3.177	2.048-2.552	1.389-1.455	
W_3N_2/G	2.144-2.461	2.439-3.023	2.051-2.412	1.377-1.471	
Nb_3N_2/NG	2.223-2.599	2.806-3.249	2.176-2.382	1.358-1.438	1.406-1.412
Ta_3N_2/NG	2.202-2.599	2.777-3.177	2.106-2.332	1.383-1.453	1.398-1.431
Mo_3N_2/NG	2.160-2.577	2.447-3.192	2.447-2.558	1.389-1.457	1.428-1.444
W_3N_2/NG	2.144-2.472	2.438-3.022	2.051-2.403	1.370-1.472	1.426-1.458

Table S8. Calculated ΔG_{H^*} values at the T_{C1}–T_{C5} and T_N sites for MXenes/G and MXenes/NG systems

**Systems**	**ΔG_{H^*} (eV)**					
	**T_{C1}**	**T_{C2}**	**T_{C3}**	**T_{C4}**	**T_{C5}**	**T_N**
Nb_3N_2/G	1.128	-0.182	0.523	0.649	0.332	---
Nb_3N_2/NG	1.182	-0.101	0.381	0.124	0.325	1.423
Ta_3N_2/G	1.138	0.243	0.581	0.330	0.179	---
Ta_3N_2/NG	1.097	0.176	0.298	0.169	0.141	1.588
Mo_3N_2/G	1.155	0.653	0.231	0.330	0.876	---
Mo_3N_2/NG	1.161	0.517	-0.005	0.083	0.786	1.051
W_3N_2/G	1.176	0.168	0.112	0.028	0.170	---
W_3N_2/NG	0.774	0.163	-0.204	-0.232	0.011	0.559

Table S9. Computed lattice constants and binding energy (E_b) for 2D composite MXenes/Si and MXenes/PSi nanostructures

**Systems**	**a (Å)**	**b(Å)**	**E_b(eV)**
Nb_3N_2/Si	11.835	11.835	31.232
Ta_3N_2/Si	11.582	11.562	33.793
Mo_3N_2/Si	11.283	11.268	32.845
W_3N_2/Si	11.072	11.028	36.532
Nb_3N_2/PSi	11.816	11.869	31.472
Ta_3N_2/PSi	11.590	11.571	33.987
Mo_3N_2/PSi	11.304	11.283	32.863
W_3N_2/PSi	11.067	11.037	35.914

Table S10. Si-TM, P-TM, TM-TM, TM-N, Si-Si, and Si-P bond lengths for 2D composite MXenes/Si and MXenes/PSi nanostructures

Systems	Si-TM(Å)	P-TM(Å)	TM-TM(Å)	TM-N(Å)	Si- Si(Å)	Si-P(Å)
Nb_3N_2/Si	2.584-3.103		2.830-3.127	2.116-2.325	2.335-2.678	
Ta_3N_2/Si	2.576-3.069		2.814-2.985	2.112-2.332	2.309-2.549	
Mo_3N_2/Si	2.471-2.915		2.668-3.170	2.063-2.367	2.277-2.418	
W_3N_2/Si	2.562-2.769		2.656-3.203	2.128-2.318	2.298-2.443	
Nb_3N_2/PSi	2.572-3.049	2.455-2.541	2.831-3.053	2.128-2.320	2.330-2.717	2.230-2.799
Ta_3N_2/PSi	2.579-3.081	2.455	2.816-2.985	2.119-2.344	2.294-2.576	2.211-2.267
Mo_3N_2/PSi	2.512-2.870	2.560	2.632-3.055	2.044-2.398	2.263-2.420	2.332-2.207
W_3N_2/PSi	2.416-2.689	2.581	2.612-2.981	2.018-2.434	2.257-2.733	2.214-2.283

Table S11. Calculated ΔG_{H^*} values at the T_{Si1}–T_{Si5} and T_P sites for MXenes/Si and MXenes/PSi systems

Systems	ΔG_{H^*} (eV)					
	T_{Si1}	T_{Si2}	T_{Si3}	T_{Si4}	T_{Si5}	T_P
Nb_3N_2/Si	-0.006	0.340	---	-0.072	0.212	---
Nb_3N_2/PSi	-0.036	0.174	0.212	0.154	0.360	---
Ta_3N_2/Si	-0.077	0.386	0.328	-0.227	0.669	---
Ta_3N_2/PSi	-0.135	0.093	0.231	0.207	---	---
Mo_3N_2/Si	0.327	0.349	0.351	-0.081	0.500	---
Mo_3N_2/PSi	0.399	0.553	0.489	-0.232	0.563	0.929
W_3N_2/Si	0.357	0.226	---	-0.097	-0.257	---
W_3N_2/PSi	-0.120	-0.446	-0.213	0.289	-0.035	0.375

Table S12. Calculated lattice constants, bond lengths, and ΔG_{H^*} values at the H_1 and H_2 sites for the sampled Nb_3N_2 supercell structure using the different k-points and truncation energies (E_{cut}).

E_{cut} (eV)	Lattice constants (Å)		Bond lengths (Å)				ΔG_{H^*} (eV)			
	3 × 3× 1	5 × 5 × 1	3 × 3× 1		5 × 5 × 1		3 × 3× 1		5 × 5 × 1	
			Nb-Nb	Nb-N	Nb-Nb	Nb-N	H_1	H_2	H_1	H_2
400	11.779	11.779	2.945	2.205-2.209	2.945	2.205-2.209	-1.106	-1.183	-1.03	-1.205
450	11.808	11.808	2.952	2.208-2.213	2.952	2.208-2.213	-1.026	-1.189	-1.056	-1.212
500	11.832	11.832	2.958	2.205-2.214	2.958	2.208-2.215	-1.023	-1.196	-1.045	-1.212
